# α-Synuclein pathology disrupts mitochondrial function in dopaminergic and cholinergic neurons at-risk in Parkinson’s disease

**DOI:** 10.1186/s13024-024-00756-2

**Published:** 2024-10-08

**Authors:** Fanni F. Geibl, Martin T. Henrich, Zhong Xie, Enrico Zampese, Jun Ueda, Tatiana Tkatch, David L. Wokosin, Elena Nasiri, Constantin A. Grotmann, Valina L. Dawson, Ted M. Dawson, Navdeep S. Chandel, Wolfgang H. Oertel, D. James Surmeier

**Affiliations:** 1 Department of Neuroscience, Feinberg School of Medicine, Northwestern University, Chicago, IL 60611 USA; 2 Department of Neurology, Philipps University Marburg, 35043 Marburg, Germany; 3 Department of Psychiatry and Psychotherapy, Philipps University Marburg, 35043 Marburg, Germany; 4 Neuroregeneration and Stem Cell Programs, Institute for Cell Engineering, Johns Hopkins University School of Medicine, Baltimore, MD 21205 USA; 5 Department of Neurology, Johns Hopkins University School of Medicine, Baltimore, MD 21205 USA; 6 Solomon H. Snyder Department of Neuroscience, Johns Hopkins University School of Medicine, Baltimore, MD 21205 USA; 7 Department of Physiology, Johns Hopkins University School of Medicine, Baltimore, MD 21205 USA; 8 Department of Pharmacology and Molecular Sciences, Johns Hopkins University School of Medicine, Baltimore, MD 21205 USA; 9 Department of Medicine, Feinberg School of Medicine, Northwestern University, Chicago, IL 60611 USA; 10 Aligning Science Across Parkinson’s (ASAP) Collaborative Research Network, Chevy Chase, MD 20815 US

**Keywords:** Parkinson’s disease, Alpha-synuclein, Mitochondria, Substantia Nigra, Dopaminergic, Pedunculopontine nucleus, Lewy pathology, Transcriptome, Electrophysiology, Bioenergetics

## Abstract

**Background:**

Pathological accumulation of aggregated α-synuclein (aSYN) is a common feature of Parkinson’s disease (PD). However, the mechanisms by which intracellular aSYN pathology contributes to dysfunction and degeneration of neurons in the brain are still unclear. A potentially relevant target of aSYN is the mitochondrion. To test this hypothesis, genetic and physiological methods were used to monitor mitochondrial function in substantia nigra pars compacta (SNc) dopaminergic and pedunculopontine nucleus (PPN) cholinergic neurons after stereotaxic injection of aSYN pre-formed fibrils (PFFs) into the mouse brain.

**Methods:**

aSYN PFFs were stereotaxically injected into the SNc or PPN of mice. Twelve weeks later, mice were studied using a combination of approaches, including immunocytochemical analysis, cell-type specific transcriptomic profiling, electron microscopy, electrophysiology and two-photon-laser-scanning microscopy of genetically encoded sensors for bioenergetic and redox status.

**Results:**

In addition to inducing a significant neuronal loss, SNc injection of PFFs induced the formation of intracellular, phosphorylated aSYN aggregates selectively in dopaminergic neurons. In these neurons, PFF-exposure decreased mitochondrial gene expression, reduced the number of mitochondria, increased oxidant stress, and profoundly disrupted mitochondrial adenosine triphosphate production. Consistent with an aSYN-induced bioenergetic deficit, the autonomous spiking of dopaminergic neurons slowed or stopped. PFFs also up-regulated lysosomal gene expression and increased lysosomal abundance, leading to the formation of Lewy-like inclusions. Similar changes were observed in PPN cholinergic neurons following aSYN PFF exposure.

**Conclusions:**

Taken together, our findings suggest that disruption of mitochondrial function, and the subsequent bioenergetic deficit, is a proximal step in the cascade of events induced by aSYN pathology leading to dysfunction and degeneration of neurons at-risk in PD.

**Supplementary Information:**

The online version contains supplementary material available at 10.1186/s13024-024-00756-2.

## Background

PD is the second most common neurodegenerative disease, afflicting millions of people worldwide. Clinically, patients manifest a combination of motor deficits, including bradykinesia, resting tremor, and/or rigidity. These symptoms are largely attributable to the degeneration of SNc dopaminergic neurons and the resulting dysregulation of motor circuits [1]. Although symptomatic treatments are available, there are no proven strategies for stopping or slowing disease progression, reflecting an incomplete grasp of PD pathogenesis.

Intraneuronal accumulation of misfolded aSYN into proteinaceous aggregates, termed Lewy pathology (LP) is a key neuropathological feature of many types of PD [2–4]. Fibrillar forms of aSYN are thought to arise *de novo* or to propagate through neuronal networks, leading to LP formation in vulnerable populations [5, 6]. Indeed, a hallmark of PD is the formation of LP in SNc dopaminergic neurons, whose degeneration is responsible for the cardinal motor symptoms of PD. However, other neurons also manifest LP in PD. For example, LP is found in PPN cholinergic neurons [6–8].

The connection between the formation of intracellular LP in neurons and neurodegeneration is still incompletely understood. The intracellular ‘interactome’ of aSYN encompasses proteins and lipids involved in many aspects of cellular function, including DNA repair, vesicular trafficking, lysosomal function, and mitochondrial metabolism [9–11]. Mitochondria are particularly intriguing members of the putative aSYN interactome because of the evidence linking them to PD pathogenesis [12]. Mitochondria in at-risk neurons have elevated oxidant stress [8, 13]. Indeed, post-mortem studies of SNc dopaminergic neurons in PD patients have revealed signs of oxidant damage to mitochondrial complex I (MCI) and mitochondrial DNA [14, 15]. Loss of MCI function alone in dopaminergic neurons is sufficient to induce a progressive parkinsonian phenotype in mice [16] and environmental toxins that disrupt MCI function have been linked to PD risk [17]. Consistent with the notion that mitochondrial damage is part of the PD etiology, loss-of-function mutations in the genes coding for PINK1 (PARK6) and Parkin (PARK2), which play important roles in mitochondrial quality control, cause early-onset PD [18, 19].

There appear to be several potential sites of interaction between aSYN-induced pathology and mitochondria, ranging from proteins at the outer mitochondrial membrane and points of contact with the endoplasmic reticulum [20, 21], to inner mitochondrial membrane complexes [22–26]. However, most of these potential sites of interaction have been identified in vitro with cell lines or immature neurons using high concentrations of aSYN, raising concerns about its relevance to pathology in an aging brain. Moreover, little of this work has focused on the types of neuron known to be selectively vulnerable to aSYN pathology in PD. This shortcoming is important because cell-type specific variation in basal stress and post-translational modifications of misfolded aSYN may be critical contributors to pathogenesis [11, 27, 28].

In an attempt to address these shortcomings, aSYN PFFs were stereotaxically injected into two PD-relevant brain regions of mice – the SNc and the PPN – and then the consequences on mitochondria were assessed using a combination of optical, transcriptomic, electrophysiological, and electron microscopic methods [7, 8]. These brain regions were selected because of their vulnerability in PD. As mentioned above, both SNc dopaminergic neurons and PPN cholinergic neurons manifest LP and are lost in PD patients [6–8]. Our studies revealed that extracellular deposition of aSYN PFFs induced cell-specific formation of intracellular aggregates containing phosphorylated aSYN (p-aSYN). This pathology disrupted mitochondrial oxidative phosphorylation, resulting in impaired mitochondrial adenosine triphosphate (ATP) generation, elevated oxidant stress, and slowing of autonomous pacemaking. In SNc dopaminergic neurons, synucleinopathy was paralleled by a complex epigenetic reprogramming, including a broad down-regulation of mitochondrial genes. In addition, aSYN PFFs induced intracellular accumulation of large end-stage lysosomes, indicative of disturbed lysosomal function. Together, these observations suggest that mitochondrial dysfunction is a proximal step in aSYN-induced neurodegeneration, pointing to a mechanistic convergence in PD pathogenesis.

## Methods

### Animals

All animal experiments were performed according to the NIH Guide for the Care and Use of Laboratory Animals and approved by the Northwestern University Animal Care and Use Committee. Mice were housed in groups of up to 5 animals per cage with food and water provided ad libitum on a 12-hour light/dark cycle. All experiments were performed in the light phase. Throughout the study, heterozygous DAT-Cre mice (B6.SJL-Slc6a3tm1.1(cre)Bkmn/J; Stock JAX:006660, Jackson Laboratory, USA) or heterozygous ChAT-Cre mice (B6;129S6-Chattm2(cre)lowl/J; Stock JAX:006410, Jackson Laboratory, USA), were used. For the electrophysiological studies, DAT-Cre or ChAT-Cre mice were crossed in-house with Ai14-tdTomato mice (B6;129S6-Gt(ROSA)26Sortm14(CAG-tdTomato)Hze/J; Stock JAX:007908, Jackson Laboratory, USA), to allow reliable cell identification. The use of DAT-Cre and ChAT-Cre lines allowed the unequivocal identification of dopaminergic and cholinergic neurons. Although there are some minor anomalies that have been found in homozygous DAT-IRES-Cre mice [29–32], all of our experimental work was done with heterozygous mice. ChAT-IRES-Cre mice are indistinguishable from wild-type mice [33]. A similar distribution of male and female mice was used in all experiments. All animals were between two and three months old at the beginning of the experiments.

### Preparation of purified aSYN PFFs and aSYN monomers

Mouse full-length aSYN PFFs were prepared at Johns Hopkins University School of Medicine, Baltimore, USA as previously described [6, 34, 35]. Briefly, purified monomeric full-length mouse aSYN was stirred at 350 rpm in a glass vial with a magnetic stir bar for 7 days. Thereafter, formed aSYN aggregates were sonicated for 30 s at 10% amplitude with a Branson Digital Sonifier (Danbury, CT, USA). Separation of aSYN monomers and PFFs was performed with fast protein liquid chromatography using a Superose 6 10/300GL column (GE Healthcare, Life Sciences, Wauwatosa, WI, USA). PFFs were briefly sonicated, and monomeric aSYN and PFFs were frozen at -80 °C. A subset of the stock solutions was then used to perform several quality control experiments, including structure analysis with transmission electron microscopy (TEM), immunoblots, and thioflavin T (ThT) binding assays. For TEM analysis, aSYN PFFs were first adsorbed to copper grids (Electron Microscopy Sciences, Hatfield, PA, USA). After washing three times, the grids were negatively stained with uranyl formate. Images were acquired using Philips/FEI BioTwin CM120 Transmission Electron Microscope (Hillsboro, OR, USA). TEM images confirmed the fibrillar morphology of PFFs (~ 100 nm) (Fig. S1a). For ThT analysis, aSYN monomers and PFFs were incubated with 50 µM ThT, and fluorescence was measured at 450 nm of excitation and 485 nm of emission wavelengths by a plate reader (Tecan, Switzerland). PFFs exhibited a significantly increased fluorescence intensity compared to aSYN monomers. The ability to induce cellular aSYN pathology was further investigated by treating primary cortical neurons from C57BL/6 mice with aSYN PFFs. After 7 days of incubation, all neurons were fixed with 4% paraformaldehyde (PFA) and permeabilized with 0.2% Triton X-100. Then, cells were blocked and incubated with primary anti-aSYN (pS129) antibody (ab51253, Abcam, Cambridge, MA, USA), confirming the induction of S129 phosphorylated aSYN pathology in primary neurons (Fig. S1b). Last, aliquoted stock solutions were shipped to Northwestern University, Chicago, USA on dry ice and stored at − 80 °C. At Northwestern University, PFFs were thawed, and sterile phosphate-buffered saline (PBS) was added to the solution to achieve a final protein concentration of 2.5 µg/µL. Thereafter, PFFs were sonicated for 90 s at 10% amplitude, aliquoted and finally stored at − 80 °C. On the day of injection, aliquoted PFFs were thawed and briefly vortexed before injection. Protocol: https://doi.org/10.17504/protocols.io.dm6gpbw28lzp/v1.

### Stereotaxic injections

All stereotaxic injections were conducted with a computer guided system (Angle Two, Leica Biosystems, USA) as previously described [6]. First, isoflurane-anesthetized mice were placed in the stereotaxic frame (David Kopf Instruments, USA). A total volume of 550 nl of PFFs or monomeric aSYN with a concentration of 2.5 µg/µl and 550 nl of the respective viral constructs was divided and injected in two injection sites for SNc and PPN and 4 injection sites for striatal injections (for SNc: AP: -3.10, ML: -1.30, DV: -4.45, and AP: -3.50, ML: -1.30, DV: -4.20; For PPN: AP: -4.36, ML: -1.12, DV: -3.70 and AP: -4.55, ML: -1.19, DV: -3.55; for STR: AP: +0.14, ML: -2.00, DV: -2.65; AP: +0.14, ML: -2.00, DV: -3.00; AP: +0.50, ML: -1.80, DV: -2.65; AP: +0.50, ML: -1.80, DV: -3.00). All injections were performed unilaterally into the left hemisphere. For SNc and PPN seeding, the injection of aSYN PFFs was intended to cover the entire brain region. For STR seeding aSYN PFFs were placed in the dorso-lateral part of the STR. To inject, a glass pipette (P-97 Pipette Puller, Sutter Instruments, Novato, CA), containing the respective viral vectors or aSYN proteins, was navigated to the injection site. After drilling a small hole, the glass pipette was slowly lowered into the brain. Injections were performed at low speed (100 nl/min) with an automated microinjector (IM-300, Narishige, Japan), and the pipette was left for an additional 5 min in the brain after the injection was completed. Protocol: https://doi.org/10.17504/protocols.io.81wgby191vpk/v1.

**Ex vivo** **brain slice preparation** Mice were anesthetized with a mixture of ketamine (50 mg/kg) and xylazine (4.5 mg/kg) and sacrificed by transcardial perfusion with ice cold, oxygenated modified artificial cerebrospinal fluid (aCSF) containing in mM: 125 sucrose, 2.5 KCl, 1.25 NaH_2_PO_4_, 25 mM NaHCO_3_, 0.5 mM CaCl_2_, 10 mM MgCl_2_, and 25 mM glucose. Once perfused, the brain was rapidly removed and coronal or parasagittal slices containing the SNc or PPN region were cut using a vibratome (VT1200S Leica Microsystems). For electrophysiological experiments, coronal SNc and parasagittal PPN slices were cut 225 μm thick, whereas for two photon laser scanning microscopy (2PLSM) experiments coronal SNc and PPN slices were cut 275 μm thick, respectively. Thereafter, brain slices were incubated in oxygenated modified aCSF containing: 135.75 mM NaCl, 2.5 mM KCl, 25 mM NaHCO_3_, 1.25 mM NaH_2_PO_4_, 2 mM CaCl_2_, 1 mM MgCl_2_, and 3.5 mM glucose; at 34 °C for 30 min, then at room temperature for another 30 min before experiments. All solutions were pH 7.4, 310–320 mOsm L^− 1^ and continuously bubbled with 95% O_2_/5% CO_2_. Experiments were performed at 32–34 °C. Protocol: 10.17504/protocols.io.x54v9p1e4g3e/v1.

### 2PLSM imaging

Fluorescence was measured using an Ultima Laser Scanning Microscope System (Prairie Technologies) with a DODT contrast detector to provide bright field transmission images, with an Olympus 60X/1.0 NA water-dipping objective lens. A Chameleon Ultra series tunable (690–1040 mm) Ti: sapphire laser system (Coherent laser group) provided the 2P excitation source. Laser power attenuation was achieved with two Pockels’ cell electro-optic modulators (M350-80-02-BK and M350-50-02-BK, Con Optics) in series controlled by PrairieView v5.3–5.5. Non-de-scanned emission photons were detected with GaAsP photomultiplier tube (PMT) (green, 490 nm to 560 nm) and multi-alkali PMT (red, 585 nm to 630 nm). All experiments were performed with a modified aCSF solution with a physiological glucose concentration (3.5 mM); glucose concentration in the cerebrospinal fluid is estimated to be between 2.8 and 4.4 mM [36]. Previous work has shown that the elevated glucose concentrations normally used to superfuse ex vivo brain slices suppress mitochondrial oxidative phosphorylation [37]. Flow rate was 2 ml/min for 2PLSM as well as electrophysiological measurements.

### 2PLSMex vivoATP/ADP measurements

Heterozygous DAT-Cre or ChAT-Cre mice were injected with either aSYN PFFs or aSYN monomer protein as described above. Ten weeks after the initial stereotactic surgery, these mice and a cohort of age- and genotype-matched mice (wild-type control) were injected with either 400 nl (SNc) or 250 nl (PPN) AAV9-EF1α-DIO-GW1-PercevalHR-WPRE (titer: 2.02 × 10^13^ vg/ml, from Virovek) into the SNc or PPN to induce expression of the genetically encoded ATP/ADP sensor PercevalHR. A small set of mice was injected 4 weeks after PFF injection to measure mitochondrial ATP production after 6 wpi. Fourteen days after AAV injection, mice were sacrificed and ex vivo brain slices were prepared as described above. Measurements were conducted as previously described [16]. Briefly, fluorescence was measured using 2PLSM as described above. To estimate the relative dependence of cellular ATP/ADP ratio on mitochondrial OXPHOS and glycolysis using PercevalHR, the probe was excited with 950 nm and 820 nm light in rapid succession. Green channel (490–560 nm) fluorescent emission signals for both wavelengths were detected using a non-descanned Hamamatsu H7422P-40 select GaAsP PMT. Two time series of 5 frames (rate of 3–4 f.p.s., 0.195 × 0.195 mm pixels and 12 µs px^− 1^ dwell time) were acquired for each wavelength. Time series were analyzed offline using FIJI. The cytosol and a background region-of-interest were measured, the background was subtracted, and the 950/820 ratio was calculated for the cytosol at each time point. MCV inhibition with oligomycin was used to estimate the contribution of OXPHOS to maintenance of cytosolic ATP/ADP ratio; 2-DG substitution for glucose in the aCSF was used to measure the contribution of glycolysis. The contribution of mitochondria to the bioenergetic status of each cell (the OXPHOS index) was estimated by comparing the drop in the PercevalHR ATP/ADP ratio induced by bath application of oligomycin (10 µM) with the drop in the ratio after superfusion with a modified aCSF with oligomycin (10 µM) and glucose replaced with the non-hydrolyzable 2-deoxy-glucose (3.5 mM).

 [38]PercevalHR measurements can be affected by intracellular pH [38]. Correcting for pH is important for the generation of an estimate of the absolute ATP/ADP ratio within a cell. In our experiments, this was not done because our primary interest was in the relative dependence of the ATP/ADP ratio on OXPHOS and glycolysis. To this end, at the initiation of recording the laser power at 820 and 950 nm was adjusted to yield a similar PercevalHR fluorescence. The changes in this fluorescence ratio were measured following application of oligomycin and 2-DG. As a consequence, our PercevalHR measurements reflect the relative dependence of cellular ATP/ADP ratio on mitochondrial OXPHOS and glycolysis and should be independent of the pH. Protocol: 10.17504/protocols.io.8epv5xe44g1b/v1.

### 2PLSMex vivoredox measurements

Oxidant stress was assessed using a redox-sensitive roGFP probe targeted to the cytosol or the mitochondrial matrix, as previously described [39]. Therefore, heterozygous DAT-Cre or ChAT-Cre mice were injected with either aSYN PFFs or aSYN monomer protein as described above. 10 weeks after the initial stereotactic surgery, those mice, and another previously not injected age-matched cohort of DAT-Cre or ChAT-Cre mice (wild-type control), were injected with either 400 nl (SNc) or 250 nl (PPN) AAV9-CMV-DIO-rev-MTS-roGFP-WPRE (titer: 2.26 × 10^13^ vg/ml, from Virovek) for expression of mitochondria-targeted roGFP, or AAV9-CMV-DIO-rev-roGFP-WPRE (titer: 2.23 × 10^13^ vg/ml, from Virovek) for expression of cytosol targeted roGFP. Five mice were injected 4 weeks post-injection to assess mitochondrial ROS production at 6 wpi. Fourteen days after AAV injection, mice were sacrificed and ex vivo brain slices were prepared as described above. Slices were transferred to a recording chamber and continuously perfused with modified aCSF at 32–34 °C at a flow rate of 2 ml/min. Fluorescence was measured using an Ultima Laser Scanning Microscope system (Bruker) with a DODT contrast detector to provide bright-field transmission images with an Olympus ×60/0.9 NA lens. A 2P laser (Chameleon Ultra II, Coherent) tuned to 920 nm was used to excite roGFP. Non-de-scanned emission photons were detected with GaAsP photomultiplier tube (PMT) from 490 nm to 560 nm. Time series images of the roGFP probe were acquired with 30 frames obtained over ~ 20 s, with 0.197 μm × 0.197 μm pixels and 10 µs dwell time. The dynamic range of the probe was determined with 2 mM dithiothreitol, a reducing agent, and 200 µM aldrithiol, an oxidizing agent, which were used to sequentially perfuse slices. Using this calibration technique, the dynamic range of the expressed fluorophore and the relative probe oxidation can then be calculated independently of the expression level of the probe [37, 39–41]. Time series images were acquired with each to determine the maximal and minimal fluorescence intensity. Time series images were analyzed offline, and fluorescence measurements in multiple regions of interest were evaluated with the background subtracted. Protocol: https://doi.org/10.17504/protocols.io.j8nlkomb1v5r/v1.

### 2PLSMex vivomeasurements of free mitochondrial Ca2+levels

Mitochondrial free Ca^2+^ levels were measured with the mitochondrially targeted Ca^2+^-sensitive probe mito-GCaMP6. Heterozygous DAT-Cre or ChAT-Cre mice were injected with either aSYN PFFs or aSYN monomer protein as described above. 10 weeks after the initial stereotactic surgery, those mice, and another previously not injected age-matched cohort of DAT-Cre or ChAT-Cre mice (wild-type control), were injected with either 400 nl (SNc) or 250 nl (PPN) AAV9-CMV-DIO-2MT-GCaMP6 (titer: 2.22 × 10^13^ vg/ml, from Virovek) for expression of mitochondrially targeted GCaMP6. 14 days after AAV injection, mice were sacrificed and ex vivo brain slices were prepared as described above. Slices were transferred to a recording chamber and continuously perfused with modified aCSF at 32–34 °C at a flow rate of 2 ml/min. Fluorescence was measured using an Ultima Laser Scanning Microscope system (Bruker) with a DODT contrast detector to provide bright-field transmission images with an Olympus ×60/0.9 NA lens. A 2P laser (Chameleon Ultra II, Coherent) tuned to 920 nm was used to excite mito-GCaMP6. Non-de-scanned emission photons were detected with GaAsP photomultiplier tube (PMT) from 490 nm to 560 nm. Time series images of the GCaMP6 probe were acquired with 30 frames obtained over ~ 20 s, with 0.197 μm × 0.197 μm pixels and 10 µs dwell time. The dynamic range of the probe was determined by the following calibration process. First, sections were perfused with a modified aCSF in which Ca^2+^ was substituted with Mg^2+^ (0 mM Ca^2+^, 3mM Mg^2+^) and the Ca^2+^ ionophore ionomycin was added at a concentration of 1 µM. Hereafter, a modified aCSF containing 3 mM Ca^2+^ and 1µM ionomycin was perfused. This calibration strategy allowed an assessment of the dynamic range of the probe that was independent of the expression level of mito-GCaMP6. Time series images were acquired with each to determine the minimal and maximal fluorescence intensity. Time series images were analyzed offline, and fluorescence measurements in multiple regions of interest were evaluated with the background subtracted. Protocol: https://doi.org/10.17504/protocols.io.5jyl8pby7g2w/v1.

### Electrophysiology

For electrophysiological experiments, DAT-Cre-Ai14-tdTomato and ChAT-Cre-Ai14-tdTomato mice were used to allow reliable identification of SNc dopaminergic or PPN cholinergic neurons. Conventional tight-seal (> 2 GΩ) patch-clamp recordings were made in cell-attached mode from either aSYN PFF or aSYN monomer injected mice, or age-matched non-injected mice. Ex vivo brain slices were prepared as described above. After slicing, 225 μm thick sections were stored in a holding chamber at room temperature filled with modified aCSF and continuously bubbled with 95% O_2_ and 5% CO_2_. Patch pipettes were pulled from thick-walled borosilicate glass pipettes on a P-1000 puller (Sutter Instrument). Pipette resistance was typically 4–5 MΩ. Recording pipettes were filled with modified aCSF. Synaptic blockers were bath-applied for all recordings (10 µM DNQX, 50 µM D-AP5, 10 µM SR 95531 hydrobromide, 1 µM CGP55845 hydrochloride). Recordings were obtained at 32–34 °C temperature, using a MultiClamp 700 A amplifier (Molecular Device) and pClamp 10 software (Molecular Device). Data were acquired at 100 kHz, filtered at 10 kHz, digitized using a DigiData 1440 (Molecular Devices), and analyzed using Clampfit 10.3 (Molecular Devices). Protocol: https://doi.org/10.17504/protocols.io.eq2lyj8zrlx9/v1.

### RiboTag profiling and RNAseq

Heterozygous DAT-Cre mice were injected with either aSYN PFFs or aSYN monomer protein into the SNc as described above. 10 weeks after the initial injection, all mice were injected with 400 nl of AAV5- EF1a-DIO-Rpl22ll-3xFlag-2 A-GFP-WPRE (Virovek; titre of 2.13 × 10^13^ vg/ml) into the SNc. Four weeks after the injection, mice were anesthetized with a mixture of ketamine (50 mg/kg) and xylazine (4.5 mg/kg) and sacrificed by transcardial perfusion with ice cold aCSF. Brains were quickly removed from the skull and 225 μm thick coronal slices containing the SNc region were cut using a vibratome (VT1200S Leica Microsystems). Thereafter, the SNc region was dissected from the tissue slices and immediately frozen at − 80 °C. RiboTag immunoprecipitation was performed as described previously [16]. In brief, tissue was homogenized in cold homogenization buffer (50 mM Tris (pH 7.4), 100 mM KCl, 10 mM MgCl_2_, 1 mM dithiothreitol, 100 µg ml^− 1^ cycloheximide, protease inhibitors and recombinant RNase inhibitors, and 1% NP-40). Homogenates were centrifuged 10,000 g for 10 min, and the resulting supernatant was precleared with protein G magnetic beads (Thermo Fisher Scientific) for 1 h at 4 °C with constant rotation. Immunoprecipitations were carried out with anti-Flag magnetic beads (Sigma-Aldrich) at 4 °C overnight with constant rotation. Four washes were carried out with high-salt buffer (50 mM Tris (pH 7.4), 350 mM KCl, 10 mM MgCl_2_, 1% NP-40, 1 mM dithiothreitol and 100 µg ml^− 1^ cycloheximide). RNA extraction was performed using the RNA-easy Micro RNA extraction kit (QIAGEN) according to the manufacturer instructions. Protocol: https://doi.org/10.17504/protocols.io.261gedwyyv47/v1.

### RNASeq analysis

RNASeq analysis was performed as previously described [16]. The quality of reads, in FASTQ format, was evaluated using FastQC. Reads were trimmed to remove Illumina adapters from the 3′ ends using cutadapt. Trimmed reads were aligned to the mus musculus genome (mm10) using STAR [42]. Read counts for each gene were calculated using htseq-count [43] in conjunction with a gene annotation file for mm10 obtained from Ensembl(http://useast.ensembl.org/index.html). Normalization and differential expression were calculated using DESeq2, which uses the Wald test [44]. The cut-off for determining significantly differentially expressed genes was a false-discovery-rate-adjusted P value of less than 0.05 using the Benjamini–Hochberg method. To reduce noise from contamination via glial and non-dopaminergic neuronal ribosomes, analysis was limited to genes expressed at least 200 counts. For pathway analysis, GSEA software (https://www.gsea-msigdb.org/gsea/index.jsp) [45] was used. Heatmaps were generated using Morpheus software (https://software.broadinstitute.org/morpheus).

### Histology and imaging for 12 wpi data

All tissue processing and immunohistochemistry was performed as previously described [6]. To achieve a sufficient fixation of the brain for further immunohistochemical analysis, all mice were anesthetized with a mixture of ketamine (50 mg/kg) and xylazine (4.5 mg/kg) and sacrificed by transcardial perfusion with ice cold 0.1 M PBS followed by 4% ice-cold PFA for 5 min. After perfusion, mice were decapitated and brains were quickly removed, followed by post-fixation for 3 days in PFA and 3 days in 30% sucrose solution in 0.1 M PBS. Brains were then frozen on dry ice and stored in -80 °C until sectioning. On the day of sectioning, brains were embedded in tissue freezing media (OCT Compound, Tissue Tek, USA) and cut into 30 μm thick consecutive coronal sections using a cryostat microtome (CM3050 S, Leica, Germany). All sections spanning the complete rostro-caudal extent of the brain were kept in correct order and stored at 4 °C in cryoprotect-solution (1:1:3 volume ratio of ethylene glycol, glycerol, and 0.1 M PB) until further processing.

Immunofluorescence staining used for data analysis or representative images were performed according to the following protocol. Sections were washed 4 × 5 min in 0.1 M PB buffer and blocked for 1 h in 10% normal donkey serum (NDS) in 0.1 M PB with 0.3% Triton X-100 (PBT) at room temperature (RT). Primary antibodies (Table S1) were diluted in 10% NDS in PBT and incubated overnight at 4 °C. On the second day, sections were washed 4 × 5 min in PBT, then incubated with fluorophore-conjugated, species-specific secondary antibodies for 2 h at RT, blocked with 10% NDS in PBT. In most cases, sections were additionally stained with DAPI (DAPI, Sigma-Aldrich, D9542-5MG, 1:10,000 of 5 mg/ml) for 10 min in 0.1 M PB. Before mounting with antifade mounting medium (ProLong Diamond Antifade Mountant, Invitrogen, P36965), sections were washed 5 × 5 min in PBT. Exceptions to this general immunofluorescence staining protocol were made for staining of S129-phosphorylated aSYN, where a streptavidin-based amplification of fluorescence was used. For this, sections were washed 4 × 5 min in 0.1 M PB, and blocked for 1 h in 10% NDS in PBT at RT. The primary antibody (anti-aSYN (pS129), Abcam, ab51253) was diluted in 10% NDS in PBT and incubated overnight at 4 °C. On the second day, after an initial wash for 4 × 5 min in PBT, sections were incubated with a biotinylated species-specific secondary antibody (biotinylated anti-rabbit, Jackson ImmunoResearch, 711-065-152, 1:1000) directed against the rabbit p-aSYN antibody with 10% NDS in PBT for 1 h at RT. Sections were then washed (3 × 5 min in PBT) and incubated with fluorophore-conjugated streptavidin (Streptavidin AlexaFluor647, Jackson ImmunoResearch, 016-600-084, 1:1000) in 10% NDS in PBT for 2 h at RT. Before mounting, sections were additionally stained with DAPI as described above and thereafter washed again 5 × 5 min in PBT. Representative fluorescent images were acquired with a TCS SP8 confocal microscope (Leica, Germany). All images were processed with FIJI to enhance signal-to-noise or to rearrange colors of certain image channels. Protocol: 10.17504/protocols.io.bp2l6xpr1lqe/v1.

### Histology for 6 wpi data

To evaluate aSYN aggregates in 6 weeks-post PFF injected mice, we injected a total volume of 550 nl of aSYN PFFs in one injection site in the left SNc (AP: -3.00, ML: -1.20, DV: -4.20). 6 weeks later, mice were anesthetized with a mixture of ketamine (50 mg/kg) and xylazine (4.5 mg/kg) and sacrificed by transcardial perfusion with saline followed by 4% PFA. After perfusion, mice were decapitated, and brains were quickly removed. Brains were cut into 80 μm thick consecutive coronal sections using a vibratome (VT1200S Leica Microsystems). For immunofluorescence staining, sections were washed 2 × 10 min in PBS buffer and blocked for 30 min in PBS with 0.1% Triton X-100 and 2% NDS at RT. Then, slices were incubated with primary antibodies (pS129, Abcam, ab51253, 1:1000; TH, Immunostar, 22941, 1:1000) diluted in PBS with 0.1% Triton X-100 and 2% NDS, and incubated overnight at 4 °C. On the second day, sections were washed 2 × 10 min in PBS, then incubated with fluorophore-conjugated, species-specific secondary antibodies (goat anti-rabbit, Invitrogen, A-11037, 1:400; goat anti-mouse, Invitrogen, A-11029, 1:400) for 30 min at RT, blocked with PBS containing 2% NDS. Before mounting with antifade mounting medium (Antifade Mounting Medium, Vector Laboratories, H-1400-10), sections were washed 1 × 15 min in PBS. Representative images were acquired with an Olympus BX41 microscope (Olympus, Japan).

### Proteinase K treatment

To analyze the formation of insoluble p-aSYN aggregates, SNc and PPN sections were digested with Proteinase K (PK) using a protocol described previously [46]. Briefly, sections containing the SNc and PPN regions were washed in 0.1 M PB and subsequently digested at 65 °C for 10 min in PBT and 12 µg/ml PK (Proteinase K, Invitrogen, #4333793). To visualize insoluble aggregates, digested sections were double stained against p-aSYN and p62 in combination with TH or ChAT, following the fluorescence staining protocol described above. Complete absence of TH and ChAT immunoreactivity indicated successful PK digestion. Sections in which TH or ChAT immunoreactivity was still visible were excluded from analysis due to incomplete PK digestion. Control sections received the same treatment without incubating them in PK. Protocol: https://doi.org/10.17504/protocols.io.81wgbx9qqlpk/v1.

### Quantification of SNc and PPN neuronal cell counts

To quantify TH-positive and NeuN-positive cells in the SNc, 30 μm thick coronal sections of 5 defined Bregma coordinates were analyzed (-2.92, -3.16, -3.40, 3.64, -3.80). To assess cell counts of ChAT-positive and NeuN-positive PPN neurons, the following 5 defined Bregma coordinates were analyzed: -4.24, -4.48, -4.72, -4.84, -4.96. First, brain tissue was washed 3 × 5 min in 0.1 M PB and thereafter quenched with 3% H2O2 and 10% methanol for 15 min at RT. After a second wash (4 × 5 min 0.1 M PB), sections were blocked for 1 h in 5% NDS in PBT. Primary antibody (anti neuronal nuclei (NeuN), Merck Millipore, MAB377, 1:1000) was diluted in 5% NDS in PBT and incubated overnight at 4 °C. On the second day, sections were washed 4 × 5 min in 0.1 M PB then incubated with a biotinylated secondary antibody (biotinylated donkey anti-mouse, Jackson ImmunoResearch, 715-065-151, 1:1000) for 1 h at RT, followed by incubation in avidin-biotin-peroxidase solution (Vectastain Elite ABC HRP Kit, Vector Laboratories, PK-6100) for 1 h at RT. Color reaction was initiated with 5% DAB (3,3’-Diaminobenzidin, Serva, Cat#18865.02), diluted in 0.1 M PB with 0.02% H_2_O_2_. After color reaction, sections were washed 4 × 5 min and blocked again for 1 h in 5% NDS in PBT. Tissue sections were then incubated with another primary antibody (for SNc sections: anti-tyrosine hydroxylase (TH), Merck Millipore, AB152, 1:1000; for PPN sections: anti-choline acetyltransferase (ChAT), Merck Millipore, AB144P, 1:100) diluted in 5% NDS in PBT overnight at 4 °C. On the third day, sections were washed 4 × 5 min in 0.1 M PB, incubated with a biotinylated secondary antibody (biotinylated donkey anti-rabbit, Jackson ImmunoResearch, 711-065-152, 1:1000; or biotinylated donkey anti-goat, Jackson ImmunoResearch, 705-065-147, 1:1000) for 1 h at RT, followed by incubation in avidin-biotin-peroxidase solution (Vectastain Elite ABC HRP Kit, Vector Laboratories, PK-6100) for 1 h at RT and initiation of color reaction with a Peroxidase Substrate Kit (SG Peroxidase Substrate Kit, Vector Laboratories, SK-4700). All stained sections were mounted, dehydrated and coverslipped with mounting medium (Eukitt medium, Sigma-Aldrich, Cat#03989). Brightfield images were acquired using an AxioImager M2 microscope (Zeiss, Germany) equipped with an Axiocam 506 color camera (Zeiss, Germany). For quantification of neuronal cell counts, the optical fractionator workflow (StereoInvestigator version 9, MicroBrightField Biosciences, USA) was used. For analysis of SNc neurons, contours were drawn based on the cytoarchitectonic distribution of TH-positive neurons, whereas for assessing PPN cell counts the distribution of ChAT-positive neurons was used. Parameters used for counting were: grid size 100 × 100 μm, counting frame 85 × 85 μm, and 2 μm guard zones. Protocol: https://doi.org/10.17504/protocols.io.e6nvwdq47lmk/v1.

### Transmission electron microscopy (TEM)

For TEM experiments, a projection-based approach was used for reliable cell identification [16]. Therefore, heterozygous DAT-Cre mice having received either aSYN PFFs or monomers 11 weeks prior, or age matched non-injected DAT-Cre mice, were injected with 400 nl of 1% Fluorogold (Santa Cruz) dissolved in saline at a rate of 100 nl per min into the dorsal striatum. The total volume was evenly distributed over four injection sites using the following Bregma coordinates: (1) AP: +0.14, ML: -2.00, DV: -2,65; (2) AP: +0.14, ML: -2.00, DV: -3.00; (3) AP: +0.50, ML: -1.80, DV: -2.65; (4) AP: +0.50, ML: -1.80, DV: -3.00. Five days later, mice were perfused at room temperature with 0.1 M PBS followed by a fixative containing 2% paraformaldehyde, 1.25% glutaraldehyde and 0.1 M phosphate buffer (pH 7.3). Thereafter, mice were decapitated, and brains quickly removed, followed by post-fixation for 24 h in the same fixative. 500 μm thick slices were cut using a vibratome and slices were visualized under the fluorescence microscope to localize the SNc, containing cells labelled with retrogradely transported Flurogold. Identified SNc region was then dissected and postfixed in buffered 2% OsO_4_, rinsed and stained in 1% uranyl acetate, dehydrated, and embedded in EMBed 812 (EMS, 14120). Ultrathin Sect. (90 nm) were contrasted with lead citrate and uranyl acetate and analyzed under a JEOL 1230 transmission electron microscope operated at 80 kV with a Gatan Orius CCD camera with the Digital Micrograph software. SNc neurons were identified by their classical morphological appearance in combination with electron dense Fluorogold particles within lysosomes [16]. The nuclei, cytoplasm, mitochondria, and lysosomes were manually outlined and analyzed using FIJI. For analysis, mitochondria were classified as healthy, swollen, or degenerated based on their morphological appearance (shape, cristae structure, intact double membrane). Protocol: https://doi.org/10.17504/protocols.io.q26g7pw88gwz/v1.

### Statistical methods

Data analysis was performed using GraphPad Prism (version 8.3.1 GraphPad Software, USA), Clampfit 10.3 (Molecular Devices), GSEA, or FIJI. In all experiments, sample size was based on prior studies using similar techniques. Sample n represents the number of neurons collected from brain slices from N animals. In our data set, the n/N ratio is always small (< 2–3), eliminating any concerns about inferred effects being skewed by unusual mice. In accordance with our previous work [37, 39, 41, 47] neurons from the same mouse were considered as independent biological replicates. Normality of the data was assessed using the Shapiro-Wilk test. When normality was inferred, parametric statistics were performed, otherwise non-parametric testing was conducted. Two-tailed tests were used unless the working hypothesis predicted a clear directionality to the change in outcome measure, in which case one-tailed tests were adopted. Data are presented using box plots showing median values, first and third quartiles, and range, unless otherwise specified. Exact statistical tests are indicated in each figure legend. Differences were considered significant at P < 0.05. Reproducibility: all key experiments were independently reproduced by different co-authors. Each experiment was performed multiple times across multiple mice as described in the figure legends. All findings in this manuscript were replicated across animals/brain slices/cells, and data were pooled for analysis and presentation. All figures were created with Adobe Illustrator version 25.1 (Adobe Systems).

## Results

### aSYN PFFs induced seeding of Lewy pathology-like inclusions and neuronal loss in SNc

**Fig. 1:**
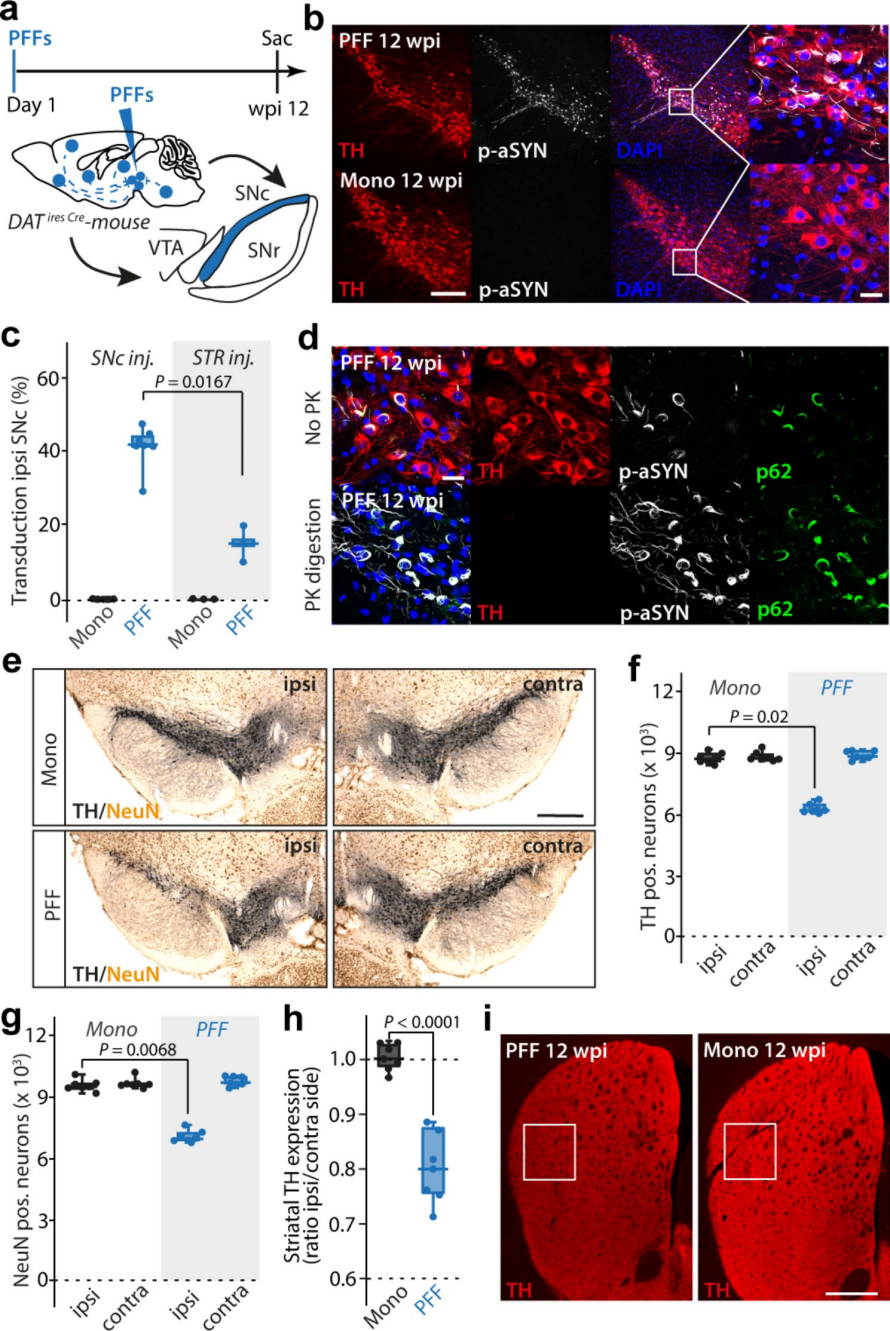
aSYN PFF injection causes PD-like neurodegeneration of midbrain DA SNc neurons. **a**, Experimental protocol. **b**, TH^+^ SNc neurons exhibiting p-aSYN pathology 12 weeks after initial seeding (upper row), while pathology is absent in aSYN monomer injected mice (lower row). Scale bar, 150 μm in overviews, 30 μm in magnified images. **c**, Transduction rates for SNc injected mice compared to conventional striatal (STR) injected mice (box plots represent median and interquartile range, whiskers min/max value; SNc (*N* = 7); STR (*N* = 3), Mann-Whitney-U test). **d**, p-aSYN^+^ aggregates were p62^+^ and resistant to digestion with Proteinase K (PK). Scale bar, 30 μm. **e**, Representative images showing neurodegeneration of DA SNc neurons on the injected side. Scale bar, 500 μm. **f**, Box plots showing number of TH^+^ neurons in SNc (box plots represent median and interquartile range, whiskers min/max value; Mono (*N* = 7), PFF (*N* = 7), Kruskal-Wallis test with Dunn’s multiple comparisons). **g**, Box plots showing number of NeuN^+^ neurons in SNc (box plots represent median and interquartile range, whiskers min/max value; Mono (*N* = 7), PFF (*N* = 7), Kruskal-Wallis test with Dunn’s multiple comparisons). **h**, Quantification of TH expression in the dorsal striatum (box plots represent median and interquartile range, whiskers min/max value; Mono (*N* = 7), PFF (*N* = 7), Unpaired t-test). **i**, Image showing TH expression in striatum. White square indicates measured ROI. Scale bar, 600 μm

As a first step towards assessing the potential impact of pathological aSYN on mitochondrial function in vulnerable neurons, mouse aSYN PFFs (or monomeric mouse aSYN) were stereotaxically injected into the SNc of mice expressing Cre recombinase (Cre) under control of the dopamine transporter (DAT) promoter (DAT-Cre) (Fig. 1a). Twelve weeks post injection (wpi), DAT-Cre mice were sacrificed, their brains fixed and sectioned for histology. At this time point, roughly 40% of tyrosine hydroxylase (TH) positive SNc dopaminergic neurons manifested immunoreactivity for aSYN phosphorylated at serine 129 (pS129) (Fig. 1b, c) – a commonly used marker of intracellular aSYN aggregation [48, 49]. Immunoreactivity for pS129 aSYN was distributed over the anterior-posterior axis of the SNc (Fig. S2a-c). In contrast, an equivalent striatal injection of aSYN PFFs induced a much lower level of SNc pS129 immunoreactivity (Fig. 1c). PFF-induced α-synucleinopathy was resistant to proteinase K (PK) digestion and immunoreactive for p62, both of which are features of human LP (Fig. 1d) [50, 51]. Unbiased stereological quantification of TH immunoreactive (TH^+^) neurons in the SNc revealed that PFFs reduced the number of TH^+^ neurons by about a third (Fig. 1e, f). To determine if the reduction in TH immunoreactivity was attributable to frank cell loss or phenotypic down-regulation, the pan-neuronal marker NeuN [52] was used to estimate the number of surviving SNc neurons. The percent reduction in NeuN^+^ neurons was similar to that of TH^+^ neurons, indicating that PFFs had induced frank neurodegeneration (Fig. 1e, g). As expected from the somatodendritic loss, axonal TH immunoreactivity in the striatum was significantly reduced (Fig. 1h, i).

Despite the spread of pS129 aSYN pathology throughout the SNc, the ventral tegmental area (VTA), and the retrorubral field (RRF), there was almost no discernible pathology in neighboring non-dopaminergic neuronal populations, like those in the substantia nigra pars reticulata (SNr) or red nucleus (RN) (Fig. S2d, e).

### aSYN pathology disrupted mitochondrial function

**Fig. 2:**
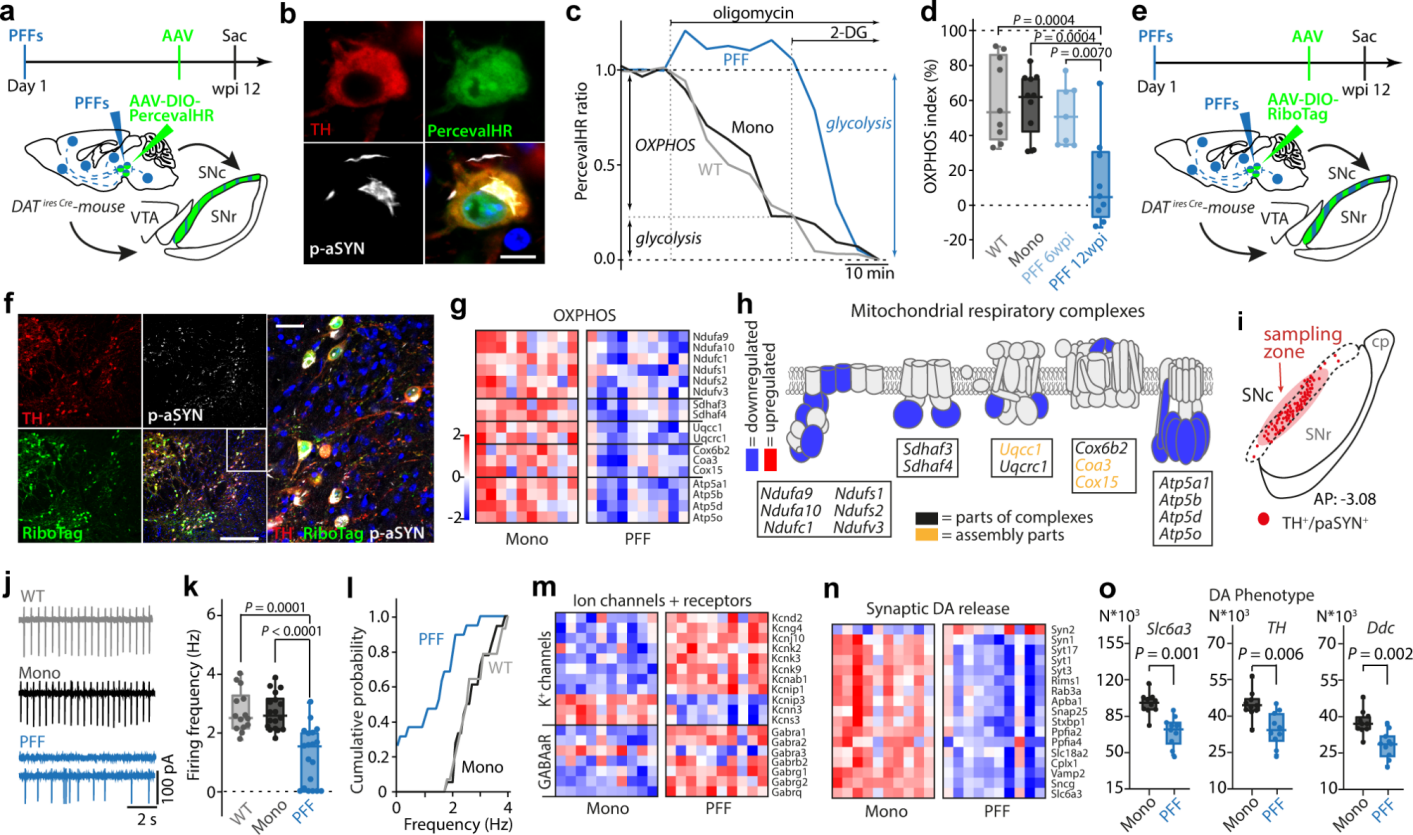
α-synucleinopathy causes disruption of mitochondrial OXPHOS resulting in energetic disbalance of DA SNc neurons. **a**, Experimental protocol for PercevalHR experiment. **b**, PercevalHR expression in a p-aSYN^+^ TH^+^ DA SNc neuron. Scale bar, 10 μm. **c**, Representative time-lapse measurements of PercevalHR fluorescence ratio. Oligomycin and 2-deoxyglucose (2-DG) were applied to determine OXPHOS and glycolytic contribution to ATP/ADP ratio. **d**, Box plots showing OXPHOS index for WT, Mono, and PFF treated mice. Synucleinopathy shifts neuronal metabolism to the glycolytic pathway at 12 wpi with mitochondria becoming net consumers of ATP. Note, at 6 wpi DA SNc neurons still possess an OXPHOS index comparable to WT and monomeric injected mice (box plots represent median and interquartile range, whiskers min/max value; WT (*N* = 8, *n* = 9), Mono (*N* = 6, *n* = 10), PFF 6 wpi (*N* = 5, *n* = 7), PFF 12 wpi (*N* = 5, *n* = 9), One-way ANOVA test with Tukey’s multiple comparisons). **e**, Experimental protocol for RiboTag experiment. **f**, Image depicting expression of RiboTag in p-aSYN^+^ DA SNc neurons. Scale bar in overviews 300 μm, and 50 μm in magnified image. **g**, Heatmap of RNASeq analysis showing significantly down- or upregulated genes of OXPHOS (Mono (*N* = 10), PFF (*N* = 10), Wald test adjusted using Benjamini-Hochberg method, *p* < 0.05). **h**, Scheme depicting significantly down- or upregulated units of the mitochondrial respiratory chain. **i**, Scheme indicating sampling zone for electrophysiological recordings within SNc. **j**, Representative cell-attached recordings of DA SNc neurons. **k**, Autonomous pacemaking of DA SNc neurons (box plots represent median and interquartile range, whiskers min/max value; WT (*N* = 5, *n* = 14), Mono (*N* = 5, *n* = 18), PFF (*N* = 5, *n* = 19), Kruskal-Wallis test with Dunn’s multiple comparisons). **l**, Cumulative probability plot of SNc DA autonomous discharge rates (WT (*N* = 5, *n* = 14), Mono (*N* = 5, *n* = 18), PFF (*N* = 5, *n* = 19). **m**,** n**, Heatmap of RNASeq analysis showing expression profiles of K^+^ channel and GABAa Receptor units (m) and downregulation of synaptic DA release genes (n) (Mono (*N* = 10), PFF (*N* = 10), Wald test adjusted using Benjamini-Hochberg method, *p* < 0.05). **o**, Box plots showing normalized gene expression values for DA phenotype genes (box plots represent median and interquartile range, whiskers min/max value; Mono (*N* = 10), PFF (*N* = 10), Wald test adjusted using Benjamini-Hochberg method, *p* < 0.05)

Misfolded forms of aSYN have been hypothesized to disrupt cellular function in a variety of ways that might ultimately lead to neurodegeneration [11]. Although mitochondrial dysfunction figures prominently in many of these hypotheses, the ability of in vivo seeding of aSYN pathology to affect them has not been systematically explored. To begin filling this gap, a combination of cellular and molecular approaches was used to study surviving SNc dopaminergic neurons in ex vivo brain slices from mice 12 wpi. To assess the ability of mitochondria to generate ATP through oxidative phosphorylation (OXPHOS), SNc dopaminergic neurons were induced to express a genetically encoded, ratiometric sensor (PercevalHR) of the cytoplasmic ATP to adenosine diphosphate (ADP) ratio [16, 38]. To this end, DAT-Cre mice were stereotaxically injected with adeno-associated virus (AAV) carrying a double-floxed inverse open reading frame (DIO) expression construct for PercevalHR 4 or 10 weeks after aSYN PFF (or monomer) injection (Fig. 2a). Histological analysis of these mice two weeks later confirmed the colocalization of TH, PercevalHR and pS129 aSYN pathology in SNc neurons (Fig. 2b). In ex vivo brain slices, 2PLSM was used to determine the PercevalHR fluorescence (following excitation with 950 and 820 nm light) and the corresponding cytosolic ATP/ADP ratio [16]. In mice without an aSYN injection (WT) or in mice with aSYN monomer (mono) injection, inhibition of mitochondrial complex V (MCV) by bath application of oligomycin resulted in a precipitous drop in cytosolic ATP/ADP ratio (Fig. 2c, d). This ratio dropped further after application of 2-DG, an inhibitor of glycolysis. These results are consistent with previous work showing that SNc dopaminergic neurons rely heavily upon mitochondrial OXPHOS to maintain cytosolic ATP levels [37]. However, by 12 wpi, the situation had changed. In SNc dopaminergic neurons from PFF injected mice, the cytosolic ATP/ADP ratio was either unaffected or actually rose following MCV inhibition, suggesting that mitochondria had ceased making a significant contribution to cytosolic ATP regeneration at this timepoint (Fig. 2c). In these neurons, the cytosolic ATP/ADP ratio had become dependent upon glycolysis, as bath application of 2-deoxyglucose (2-DG) caused a profound drop in the PercevalHR ratio (Fig. 2c, d).

To gain a better understanding of the mechanisms responsible for this metabolic shift, two additional experiments were performed. First, a cohort of mice was sacrificed 6 weeks after PFF injection for analysis of the mesencephalon. In these mice, there was no discernible cell loss, however the percentage of SNc dopaminergic neurons manifesting pS129 immunoreactivity was similar to that at 12 wpi (~ 30–40%; Fig. S3a, b). Many dopaminergic neurons had robust perinuclear pS129 staining, but many also had more punctate staining. Interestingly, mitochondrial ATP production in these dopaminergic neurons, as assessed using PercevalHR, was relatively normal, in contrast to the situation at 12 wpi (Fig. 2d). Second, actively transcribed messenger ribonucleic acids (mRNAs) were harvested from SNc dopaminergic neurons in aSYN monomer or PFF-injected mice 12 wpi and then subjected to RNASeq analysis [53]. To selectively harvest mRNAs from dopaminergic neurons, DAT-Cre mice were stereotaxically injected with an AAV vector carrying a DIO-RiboTag expression construct (Fig. 2e). Histological analysis of these mice confirmed the robust co-localization of TH, pS129 aSYN and the RiboTag reporter (Fig. 2f). Consistent with the functional studies, gene set enrichment analysis (GSEA) (Fig. S4a, b) revealed a broad down-regulation in the expression of genes coding for OXPHOS-related proteins in dopaminergic neurons from aSYN PFF injected mice (Fig. 2g, h; Fig. S4c). Although this feature of the translatomes of dopaminergic neurons resembled that seen following loss of MCI function by deletion of *Ndufs2* [16], other features differed. Of particular note, there was no up-regulation of genes coding for proteins participating in glycolysis, and genes associated with lactate metabolism were down-regulated (Fig. S4c, d). Interestingly, genes associated with beta-oxidation of fatty acids and ketone body consumption were upregulated (Fig. S4c, d). In addition to the reprogramming of metabolic pathways, GSEA revealed upregulation of apoptosis-related genes and genes linked to hypoxia (Fig. S4e, f). Ex vivo electrophysiological examination of SNc dopaminergic neurons revealed that PFF exposure suppressed autonomous pacemaking (Fig. 2i-l). On the transcriptomic level, these electrophysiological changes were associated with an up-regulation of genes coding for plasma membrane K^+^ channels and GABA_A_ receptors (Fig. 2m; Fig. S4f) and down-regulation of genes associated with the synthesis, synaptic release and uptake of dopamine (*Slc6a3*,* Th*,* Ddc*) (Fig. 2n, o).

### aSYN PFFs elevated oxidant stress, lowered mitochondrial mass, and induced lysosomal pathology

**Fig. 3:**
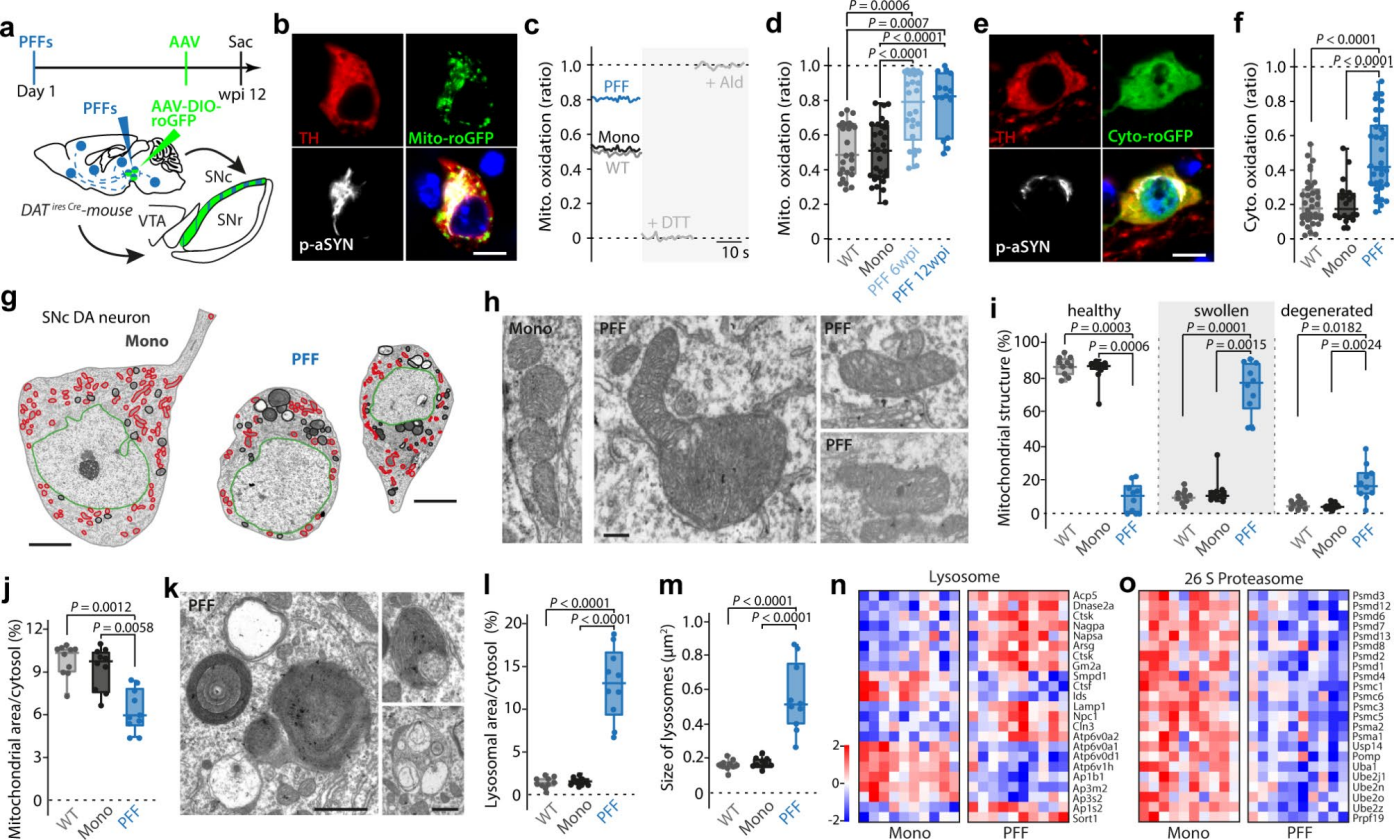
α-synucleinopathy leads to mitochondrial oxidation and morphological alterations of mitochondria and lysosomes. **a**, Experimental protocol. **b**, Mito-roGFP expression in a p-aSYN^+^ TH^+^ DA SNc neuron. Scale bar, 10 μm. **c**, Calibration protocol. **d**, Synucleinopathy elevates mitochondrial ROS levels already at 6 wpi (box plots represent median and interquartile range, whiskers min/max value; WT (*N* = 5, *n* = 26), Mono (*N* = 5, *n* = 30), PFF 6 wpi (*N* = 6, *n* = 26), PFF 12 wpi (*N* = 5, *n* = 15), Kruskal-Wallis test with Dunn’s multiple comparisons). **e**, Cyto-roGFP expression in a p-aSYN^+^ TH^+^ DA SNc neuron. Scale bar, 10 μm. **f**, Synucleinopathy increases basal cytosolic oxidation (box plots represent median and interquartile range, whiskers min/max value; WT (*N* = 5, *n* = 34), Mono (*N* = 5, *n* = 20), PFF (*N* = 5, *n* = 36), Kruskal-Wallis test with Dunn’s multiple comparisons). **g**, Transmission electron micrographs of SNc DA neurons from monomeric aSYN (left) or PFF (right) injected mice. The nucleus is highlighted in green, mitochondria in red, and lysosomes in black, respectively. Scale bar 10 μm. **h**, Series of transmission electron micrographs showing healthy, swollen, and degenerated mitochondria of SNc DA neurons. Scale bar 300 nm. **i**, Box plots showing quantification of mitochondrial morphology (box plots represent median and interquartile range, whiskers min/max value; WT (*N* = 4, *n* = 10), Mono (*N* = 4, *n* = 10), PFF (*N* = 4, *n* = 10), Kruskal-Wallis test with Dunn’s multiple comparisons). **j**, Box plots indicating mitochondrial density as percent of cytosol area (box plots represent median and interquartile range, whiskers min/max value; WT (*N* = 4, *n* = 10), Mono (*N* = 4, *n* = 10), PFF (*N* = 4, *n* = 10), Kruskal-Wallis test with Dunn’s multiple comparisons). **k**, Transmission electron micrographs showing different lysosome stages, including multilamellar bodies, in a SNc DA neuron from a PFF injected mouse. Scale bar 1 μm. **l**, Quantification of lysosome density as percent of cytosol area (box plots represent median and interquartile range, whiskers min/max value; WT (*N* = 4, *n* = 10), Mono (*N* = 4, *n* = 10), PFF (*N* = 4, *n* = 10), One-way ANOVA test with Tukey’s multiple comparisons). **m**, Box plots indicating average size of lysosomes (box plots represent median and interquartile range, whiskers min/max value; WT (*N* = 4, *n* = 10), Mono (*N* = 4, *n* = 10), PFF (*N* = 4, *n* = 10), One-way ANOVA test with Tukey’s multiple comparisons). **n**,** o**, Heatmaps of RNASeq analysis showing significantly down- or upregulated genes of lysosomal (n), and proteasomal (o) degradation pathways (Mono (*N* = 10), PFF (*N* = 10), Wald test adjusted using Benjamini-Hochberg method, *p* < 0.05)

To assess the impact of aSYN PFF-induced pathology on mitochondrial and cytosolic redox status, genetically encoded redox-sensitive variants of green fluorescent protein (roGFP) were targeted either to the mitochondrial matrix (mito-roGFP) or the cytosol (cyto-roGFP) [39]. Four or ten weeks after the initial delivery of aSYN PFFs or monomers, DAT-Cre mice underwent a second stereotaxic surgery in which an AAV carrying a DIO mito-roGFP or cyto-roGFP expression construct was injected into the SNc (Fig. 3a). Histological analysis of these mice two weeks later confirmed the colocalization of TH, mito-roGFP and pS129 aSYN (Fig. 3b). Mitochondrial targeting of the roGFP probe was confirmed by immunohistochemistry, which demonstrated colocalization of the mitochondrial marker TOMM20 and mito-roGFP in TH^+^ dopaminergic SNc neurons (Fig. S5a). 2PLSM was then used in ex vivo brain slices to measure mitochondrial oxidant stress (Fig. 3c). These experiments revealed that mitochondrial oxidant stress was elevated in SNc dopaminergic neurons 6 weeks following aSYN PFF exposure but not following exposure to monomers. Mitochondrial oxidant stress remained elevated at 12 wpi (Fig. 3c, d). The elevation was not attributable to increased mitochondrial Ca^2+^ loading, as matrix Ca^2+^ concentration measured with mito-GCaMP6 did not appear to be altered by PFFs (Fig. S6a-c). Consistent with previous work in vitro [54], aSYN PFFs also elevated cytosolic oxidant stress (measured with the cyto-roGFP probe) (Fig. 3e, f). On the transcriptomic level, these alterations were paralleled by an upregulation of *ATF5* (Fig. S6e), a key mediator of the mitochondrial unfolded protein response (mUPR) [55].

Oxidant stress can damage proteins, lipids, and deoxyribonucleic acid (DNA). To assess the structural impact of sustained oxidant stress on SNc dopaminergic neurons, they were retrogradely labeled by striatal injection of Fluorogold, and then mice were sacrificed and processed for transmission electron microscopy (TEM) (Fig. S7a). aSYN PFF exposure reduced the somatic cross-sectional area of SNc dopaminergic neurons (Fig. 3g; Fig. S7b, c). Mitochondria in PFF-exposed neurons commonly were dysmorphic and had altered cristae structure (Fig. 3h, i; Fig. S7d; Fig. S8). Dysmorphic mitochondria were distributed throughout the cytosol. Mitochondrial density and the total cytosolic area occupied by mitochondria were significantly reduced by aSYN PFFs, whereas mitochondrial size remained unaltered (Fig. 3j; Fig. S7e, f). SNc dopaminergic neurons from monomer or non-injected DAT-Cre-WT mice did not display any of these features (Fig. S8).

Exposure to aSYN PFFs also increased the proportion of the cytosol occupied by lysosomes, which was largely attributable to increased lysosomal size (Fig. 3k-m, Fig. S7g, h). The perinuclear region of PFF-exposed SNc dopaminergic neurons was crowded with endolysosomes, autophagosomes, and terminal lysosomes having a characteristic multilamellar appearance (Fig. 3k; Fig. S7i; Fig. S8). Occasionally, mitochondria were found in these multilamellar bodies (Fig. S7j). The aSYN PFF-induced lysosomal adaptation was accompanied by a complex alteration in gene expression. Genes coding for lysosomal enzymes and structural proteins were upregulated, but genes coding for ATPases and adapter proteins were down-regulated (Fig. 3n; Fig. S7k). Also, genes coding for proteasomal proteins were broadly down-regulated (Fig. 3o, Fig. S7k), in agreement with previous reports of compromised proteasomal capacity in the presence of synuclein pathology [56, 57].

### aSYN PFFs induced similar bioenergetic changes in PPN cholinergic neurons

**Fig. 4:**
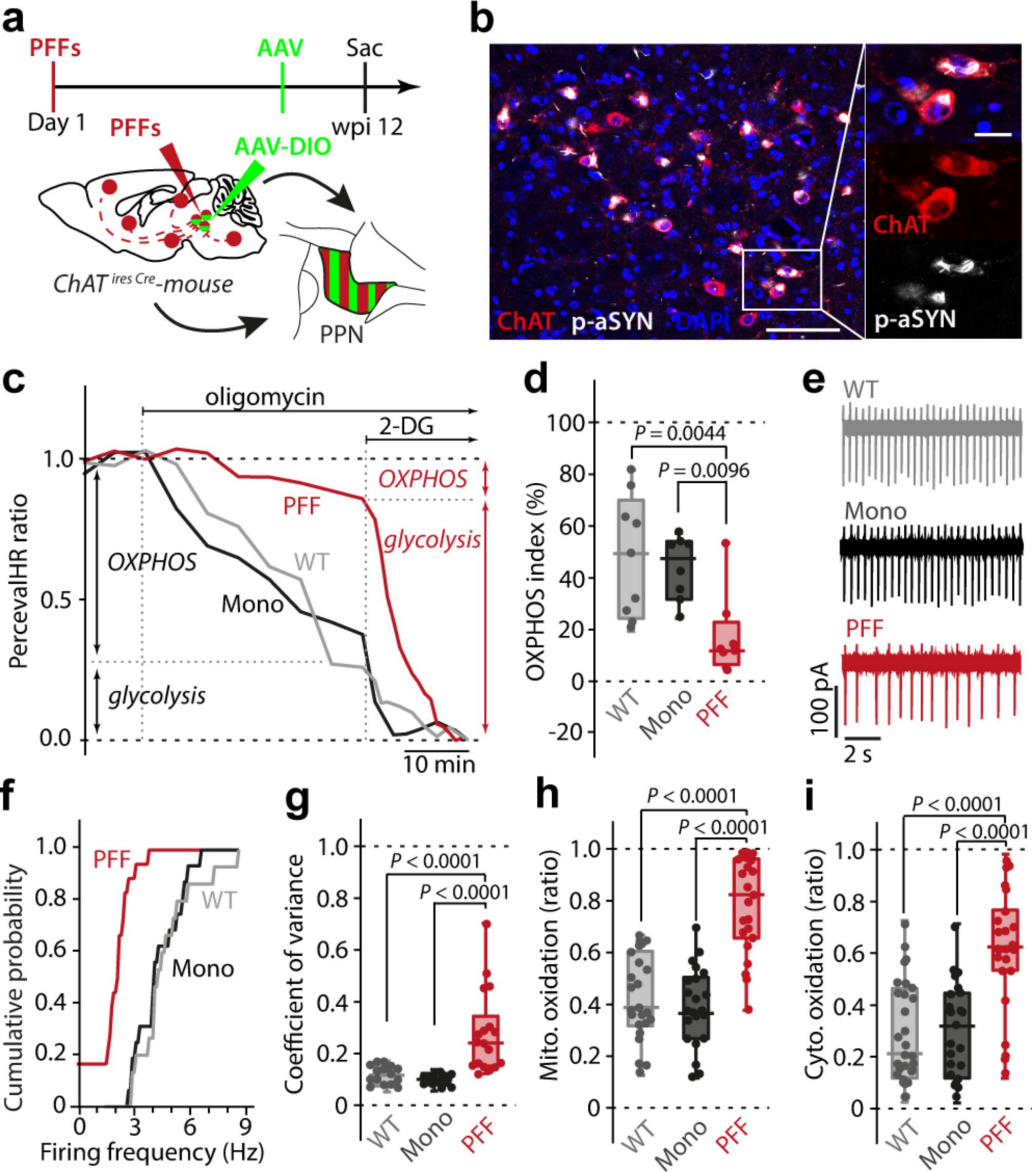
α-synucleinopathy induces similar metabolic, electrophysiological, and oxidative alterations in cholinergic PPN neurons. **a**, Experimental protocol. **b**, Image depicting p-aSYN pathology in ChAT^+^ PPN neurons 12 weeks after initial seeding. Scale bar, 100 μm in overview, 20 μm in magnified image. **c**, Representative time-lapse measurements of PercevalHR fluorescence ratio. Oligomycin and 2-DG were applied to determine OXPHOS and glycolytic contribution to ATP/ADP ratio. **d**, Box plots showing OXPHOS index for WT, Mono, and PFF treated mice. Synucleinopathy shifts neuronal metabolism to the glycolytic pathway (box plots represent median and interquartile range, whiskers min/max value; WT (*N* = 7, *n* = 8), Mono (*N* = 7, *n* = 8), PFF (*N* = 6, *n* = 9), Kruskal-Wallis test with Dunn’s multiple comparisons). **e**, Representative cell-attached recordings of identified ChAT^+^ PPN neurons. **f**, Cumulative probability plots of ChAT^+^ PPN autonomous discharge rates (WT (*N* = 6, *n* = 15), Mono (*N* = 5, *n* = 16), PFF (*N* = 6, *n* = 18). **g**, Box plots showing coefficient of variance (box plots represent median and interquartile range, whiskers min/max value; WT (*N* = 6, *n* = 15), Mono (*N* = 5, *n* = 16), PFF (*N* = 6, *n* = 18), Kruskal-Wallis test with Dunn’s multiple comparisons). **h**, Basal mitochondrial oxidative stress is elevated in cholinergic PPN neurons of PFF injected mice (box plots represent median and interquartile range, whiskers min/max value; WT (*N* = 7, *n* = 23), Mono (*N* = 5, *n* = 22), PFF (*N* = 6, *n* = 23), Kruskal-Wallis test with Dunn’s multiple comparisons). **i**, Basal cytosolic ROS levels are significantly increased in cholinergic PPN neurons of PFF injected mice (box plots represent median and interquartile range, whiskers min/max value; WT (*N* = 5, *n* = 28), Mono (*N* = 5, *n* = 23), PFF (*N* = 5, *n* = 24), Kruskal-Wallis test with Dunn’s multiple comparisons) **Declarations**

Previous studies have suggested that dopaminergic neurons were at elevated risk of neurodegeneration in PD because of an interaction between oxidized forms of dopamine and aSYN [13, 58]. However, aSYN pathology in the brains of PD patients is found in a variety of non-dopaminergic neurons that ultimately degenerate. For example, cholinergic neurons in the brainstem PPN not only manifest LP in PD but are lost in the course of the disease, much like SNc dopaminergic neurons [7, 59]. To determine if seeded α-synucleinopathy induced a similar set of bioenergetic alterations in these neurons, aSYN PFFs were injected into the PPN of mice expressing Cre recombinase under the control of the promoter for choline acetyltransferase (ChAT) to allow cell-specific measurements to be performed (ChAT-Cre) (Fig. 4a; Fig. S9a). Twelve weeks after seeding, the majority of PPN cholinergic neurons manifested p-aSYN pathology that was PK resistant (Fig. 4b; Fig. S9b, c,i). As previously shown [6], p-aSYN pathology was almost exclusively found in the cholinergic PPN neurons, being largely absent in neighboring glutamatergic and GABAergic neurons (Fig. S9d, e). Unbiased stereological counts of ChAT^+^ immunoreactive and NeuN^+^ PPN neurons confirmed that aSYN PFF exposure induced significant neurodegeneration (Fig. S9f-h). Mitochondrial function in surviving PPN cholinergic neurons was assessed using the same strategy described above employing genetically encoded biosensors (Fig. 4a, b). These experiments revealed that aSYN PFF exposure compromised the ability of mitochondrial OXPHOS to generate ATP (Fig. 4c, d). This change was accompanied by alterations of PPN cholinergic neurons electrophysiological properties, including slowing of pacemaking and an increase in irregularity of discharge (Fig. 4e-g). As in dopaminergic SNc neurons, pathological aSYN caused increased levels of mitochondrial and cytosolic oxidant stress in PPN cholinergic neurons (Fig. 4h, i). Taken together, these results suggest that PFFs induce a qualitatively similar set of deficits in dopaminergic and cholinergic neurons at-risk in PD.

## Discussion

Our studies provide new insight into the cascade of events triggered by synucleinopathy that lead to neuronal dysfunction and degeneration. Twelve weeks after stereotaxic injection of mouse aSYN PFFs in the SNc, roughly a third of the dopaminergic neurons near the injection site had degenerated, and nearly half of the surviving dopaminergic neurons had clearly discernible intracellular aggregates containing PK-resistant pS129 aSYN, as well as p62 – all hallmarks of LP [48, 51]. This pathology was limited to PD-vulnerable dopaminergic neurons in the region, leaving neighboring non-dopaminergic neurons of the SNr and RN ostensibly unaffected. The appearance of intracellular aSYN pathology was accompanied by a profound disruption in mitochondrial function, as well as clear signs of lysosomal stress. Similar changes were seen in vulnerable PPN cholinergic neurons following PFF seeding.

### aSYN PFF seeding induced cell-specific pathology

In PD, SNc dopaminergic neurons are selectively vulnerable to LP and degeneration. Neighboring neurons in the SNr, zona incerta, and RN are devoid of LP [8]. This selective pattern of pathology was recapitulated three months after stereotaxic injection of aSYN PFFs into the SNc. At this time, roughly a third of SNc dopaminergic neurons had been lost. Interestingly, cell counts based on TH and NeuN expression yielded very similar numbers, suggesting that PFFs did not trigger senescence, at least not in a substantial population of SNc dopaminergic neurons [60]. In the surviving dopaminergic neurons, nearly half had perinuclear PK-resistant inclusions that were immunoreactive for pS129 aSYN and p62. This pathology was found throughout the rostro-caudal and medio-lateral extent of the SNc and in the VTA, but rarely in neighboring structures. A similar degree of selectivity was found following aSYN PFF injection into the PPN, where PPN cholinergic neurons manifested pS129 aSYN pathology, but not neighboring glutamatergic or GABAergic neurons [6].

Why there was cellular specificity in the pathology induced by aSYN PFFs is unclear. A variety of mechanisms have been proposed to mediate uptake of aSYN fibrils from the extracellular space [61]. But essentially all of these mechanisms, like macropinocytosis, are ones that are common to most (if not all) cell types. Recent work has pointed to the importance of intracellular Ca^2+^ signaling in macropinocytosis and the uptake of aSYN PFF [62, 63]. Indeed, robust intracellular Ca^2+^ signaling and weak intrinsic Ca^2+^ buffering are features that distinguish SNc dopaminergic neurons from neighboring SNr GABAergic neurons [64–66]. Heterogeneity in Ca^2+^ signaling and linked endocytic processes also might be responsible for the apparent resistance of many SNc dopaminergic neurons to aSYN PFF seeding [67].

However, it is important to note that phosphorylation of aSYN at S129 is a relatively late event in the cascade leading to intracellular inclusions [49]. As a consequence, it could be that mesencephalic neurons indiscriminately take up aSYN PFFs, but some nominally resistant neurons dispose of them before they escape into the cytoplasm, aggregate, and are phosphorylated. It is also possible that the selective distribution of pathology seen in our experiments was dependent upon the timing of the assay (12 wpi) and the species of aSYN PFFs used [35, 68]. Nevertheless, the selectivity of the observed pathology argues that cell autonomous factors contribute in a significant way to the distribution of aSYN pathology observed in PD.

### aSYN PFFs induced a complex intracellular pathology

The intracellular pathology induced in dopaminergic neurons by PFFs was varied in composition and structure. EM examination of retrogradely labeled dopaminergic neurons revealed shrunken somata and an increase in the proportion of the cytoplasm occupied by endolysosomes, autophagosomes, and multilamellar terminal lysosomes. Often, these inclusions were found in the perinuclear region of the cell and contained fragments of mitochondria, like some types of LP [69]. However, dysmorphic mitochondria were observed throughout the cytosol, not just in the perinuclear region. As one might expect from this pathology, there was a concomitant up-regulation in the expression of genes associated with lysosomal function in dopaminergic neurons as assessed using the RiboTag method to isolate cell-specific mRNAs [16]. Although there was an alignment with the lysosomal morphology, there are caveats to the transcriptomic studies. One is that the isolation of tagged ribosomes is not perfect, leading to some degree of contamination from other cell types, like astrocytes and microglia, which clearly respond to aSYN PFF seeding [70]. To limit the impact of this shortcoming on the pathway analysis, only mRNAs with counts above 200 were included. Another consideration is that not all of the dopaminergic neurons manifested aSYN pathology, leading to a mix of mRNAs from ostensibly challenged and unchallenged neurons. As a consequence, our profiling may have under-estimated the magnitude of the transcriptome changes induced by aSYN PFFs. This heterogeneity could explain some puzzling features of the profiles, like the apparent down-regulation in mRNAs for subunits of the lysosomal V-ATPase. It is also possible that this change reflects the suppression of neurotransmission, which relies upon vesicular V-ATPase for sequestration of dopamine, rather than compromised lysosomal function. Interestingly, while the expression of lysosomal genes was increased by PFF exposure, those linked to the proteasome were down-regulated, in agreement with previous work [56, 57].

### aSYN PFFs disrupted mitochondrial function

Perhaps the most profound change observed in dopaminergic neurons following aSYN PFF seeding was the disruption of mitochondrial OXPHOS. Based upon PercevalHR measurements, at 12 wpi, mitochondria in inclusion-bearing neurons made little, if any, contribution to maintaining the cytosolic ATP/ADP ratio, in contrast to control or monomer-exposed neurons. In fact, in many neurons, cytosolic ATP/ADP ratio rose following inhibition of MCV with oligomycin, indicating that mitochondria were importing ATP from the cytosol to stay polarized. Consistent with this functional assay, PFF exposure down-regulated the expression of a wide array of somatic genes coding for OXPHOS proteins and suppressed autonomous spiking [71], a consequence attributable to an up-regulation in K^+^ channels. Paralleling these changes in activity, the expression of genes related to dopamine synthesis, sequestration, and release were also down-regulated by aSYN PFF seeding.

In several respects, these sequelae were like those induced by targeted genetic disruption of MCI function, which leads to a slow, progressive degeneration of SNc dopaminergic neurons and a parkinsonian phenotype in mice [27]. However, there were several significant differences. One difference was that aSYN PFF seeding ostensibly failed to up-regulate the expression of genes coding for proteins involved in glycolysis to compensate for the loss of mitochondrial OXPHOS. It is possible that in those dopaminergic neurons with aSYN pathology, there was an up-regulation in glycolytic genes, but this change was obscured by the inclusion of dopaminergic neurons in the RiboTag sample that did not have aSYN pathology. Another possibility is that the aSYN PFF-induced metabolic remodeling in dopaminergic neurons was constrained by the pleiomorphic nature of the synuclein insult. A key node in the signaling pathway mediating metabolic adaptations is the mammalian target of rapamycin complex 1 (mTORC1) [72]. Recent work has revealed that aSYN fibrils stimulate aberrant mTORC1 activity by binding to tuberous sclerosis protein 2 [73]. In other neurons, mTORC1 up-regulates glycolysis following disruption of mitochondrial electron transport function [74]. Thus, elevated mTORC1 activity could be responsible for the enhanced glycolytic activity in PFF-seeded dopaminergic neurons, independently of any alteration in gene transcription. This metabolic shift also might redirect glycolytic flux away from the pentose phosphate pathway and nicotinamide adenine dinucleotide phosphate generation [75]– compromising cytosolic oxidant defenses and inducing the elevation in cytosolic oxidant stress seen in dopaminergic neurons following aSYN PFF seeding.

Another unresolved question is the nature of the interaction between cytosolic aSYN fibrils and mitochondria. That is, how do aSYN fibrils bring about a deficit in mitochondrial OXPHOS? The physical dimensions of aSYN fibrils precludes the possibility that they are entering mitochondria through outer membrane pores or transporters [59]. Although aSYN fibrils can disrupt mitochondrial membranes in some circumstances [26, 76], this should trigger cytochrome C release, apoptosis, and rapid cell death, which is not what appears to be happening in most dopaminergic neurons in vivo following aSYN PFF seeding. Another possibility is that the effect of cytosolic aSYN fibrils on mitochondria is secondary to lysosomal or autophagic dysfunction. Lysosomal pathology was a prominent feature of dopaminergic neurons following seeding. It is reasonable to assume that the transit of fibrils from the endolysosomal space into the cytosol reflects lysosomal leakage (at least in part) [60]. As the lysosomal compartment is enriched in Ca^2+^, iron, and proteolytic enzymes, the loss of lysosomal integrity could have direct, deleterious consequences on mitochondria, beyond those mediated by perturbation in mTOR activity and mitochondrial quality control [77]. However, six weeks after PFF injection, when there was a similar level of pS129 immunoreactivity in SNc dopaminergic neurons (and hence transit of fibrils from the endolysosomal space to the cytosol), mitochondrial ATP production appeared to be intact. This suggests that some aspect of the cellular response to cytosolic fibrils, perhaps some early step in the formation of Lewy pathology, triggers mitochondrial dysfunction rather than fibrils themselves [27]. Additional longitudinal studies will be necessary to rigorously determine what these factors might be, how they drive mitochondrial dysfunction and how mitochondrial dysfunction contributes to neurodegeneration.

## Conclusions

Mitochondrial dysfunction and aSYN-enriched LP are hallmarks of PD. But, whether the two are mechanistically linked in a disease relevant setting has been unclear. The studies presented here show that in vivo exposure to aSYN PFFs leads to profound, complex intracellular pathology that includes mitochondrial dysfunction in two types of neurons at risk in PD – SNc dopaminergic neurons and PPN cholinergic neurons. These studies point to a pivotal role for mitochondrial dysfunction in aSYN-induced neuronal dysfunction and degeneration, underscoring the potential therapeutic benefit of boosting mitochondrial function in early-stage PD patients [12].

## Electronic supplementary material

Below is the link to the electronic supplementary material.

Supplementary Material 1

## Data Availability

The datasets generated and/or analyzed during the current study are available in the Zenodo repository (DOI: 10.5281/zenodo.13837652)

## Abbreviations

2-DG: 2-deoxyglucose
2PLSM: Two photon laser scanning microscopy
AAV: Adeno-associated virus
aCSF: Artificial cerebrospinal fluid
ADP: Adenosine diphosphate
aSYN: Alpha-synuclein
ATP: Adenosine triphosphate
ChAT: Choline acetyltransferase
Cre: Cre recombinase
cyto-roGFP: RoGFP targeted to cytosol
DAT: Dopamine transporter
DIO: Double-floxed inverse open reading frame
DNA: Deoxyribonucleic acid
ETC: Electron transport chain
GABA_A_: Gamma-aminobutyric acid type A
GSEA: Gene set enrichment analysis
ISR: Induced stress response
LP: Lewy pathology
MCI: Mitochondrial complex I
MCV: Mitochondrial complex V
mito-roGFP: RoGFP targeted to mitochondrial matrix
mRNAs: Messenger ribonucleic acids
mTORC1: Mammalian target of rapamycin complex 1
NADH: Nicotinamide adenine dinucleotide + hydrogen
OXPHOS: Oxidative phosphorylation
PBS: Phosphate-buffered saline
PD: Parkinson’s disease
PFA: Paraformaldehyde
PFFs: Pre-formed alpha-synuclein fibrils
PINK1: PTEN induced kinase 1
PK: Proteinase K
PPN: Pedunculopontine nucleus
pS129: Phosphorylation at serine 129
roGFP: Redox-sensitive variant of green fluorescent protein
SNc: Substantia nigra pars compacta
SNr: Substantia nigra pars reticulata
TEM: Transmission electron microscopy
TH: Tyrosine hydroxylase
ThT: Thioflavin T
WT: Wildtype

## References

[1] Poewe W, Seppi K, Tanner CM, Halliday GM, Brundin P, Volkmann J, Schrag AE, Lang AE. Parkinson disease. Nat Rev Dis Primers. 2017;3:17013. 28332488 10.1038/nrdp.2017.13

[2] Singleton AB, Farrer M, Johnson J, Singleton A, Hague S, Kachergus J, Hulihan M, Peuralinna T, Dutra A, Nussbaum R, et al. Alpha-synuclein locus triplication causes Parkinson’s disease. Science. 2003;302:841. 14593171 10.1126/science.1090278

[3] Spillantini MG, Schmidt ML, Lee VM, Trojanowski JQ, Jakes R, Goedert M. Alpha-synuclein in Lewy bodies. Nature. 1997;388:839–40. 9278044 10.1038/42166

[4] Braak H, Del Tredici K, Rub U, de Vos RA, Jansen Steur EN, Braak E. Staging of brain pathology related to sporadic Parkinson’s disease. Neurobiol Aging. 2003;24:197–211. 12498954 10.1016/s0197-4580(02)00065-9

[5] Luk KC, Kehm V, Carroll J, Zhang B, O’Brien P, Trojanowski JQ, Lee VM. Pathological alpha-synuclein transmission initiates Parkinson-like neurodegeneration in nontransgenic mice. Science. 2012;338:949–53. 23161999 10.1126/science.1227157 PMC3552321

[6] Henrich MT, Geibl FF, Lakshminarasimhan H, Stegmann A, Giasson BI, Mao X, Dawson VL, Dawson TM, Oertel WH, Surmeier DJ. Determinants of seeding and spreading of alpha-synuclein pathology in the brain. Sci Adv 2020, 6. 10.1126/sciadv.abc2487 PMC7673735 33177086

[7] Giguere N, Burke Nanni S, Trudeau LE. On cell loss and selective vulnerability of neuronal populations in Parkinson’s Disease. Front Neurol. 2018;9:455. 29971039 10.3389/fneur.2018.00455 PMC6018545

[8] Surmeier DJ, Obeso JA, Halliday GM. Selective neuronal vulnerability in Parkinson disease. Nat Rev Neurosci. 2017;18:101–13. 28104909 10.1038/nrn.2016.178 PMC5564322

[9] Lassen LB, Reimer L, Ferreira N, Betzer C, Jensen PH. Protein Partners of Alpha-Synuclein in Health and Disease. Brain Pathol. 2016;26:389–97. 26940507 10.1111/bpa.12374 PMC8029326

[10] Minakaki G, Krainc D, Burbulla LF. The convergence of Alpha-Synuclein, mitochondrial, and lysosomal pathways in vulnerability of midbrain dopaminergic neurons in Parkinson’s Disease. Front Cell Dev Biol. 2020;8:580634. 33381501 10.3389/fcell.2020.580634 PMC7767856

[11] Wong YC, Krainc D. Alpha-synuclein toxicity in neurodegeneration: mechanism and therapeutic strategies. Nat Med. 2017;23:1–13. 28170377 10.1038/nm.4269 PMC8480197

[12] Henrich MT, Oertel WH, Surmeier DJ, Geibl FF. Mitochondrial dysfunction in Parkinson’s disease - a key disease hallmark with therapeutic potential. Mol Neurodegener. 2023;18:83. 37951933 10.1186/s13024-023-00676-7 PMC10640762

[13] Burbulla LF, Song P, Mazzulli JR, Zampese E, Wong YC, Jeon S, Santos DP, Blanz J, Obermaier CD, Strojny C, et al. Dopamine oxidation mediates mitochondrial and lysosomal dysfunction in Parkinson’s disease. Science. 2017;357:1255–61. 28882997 10.1126/science.aam9080 PMC6021018

[14] Flones IH, Fernandez-Vizarra E, Lykouri M, Brakedal B, Skeie GO, Miletic H, Lilleng PK, Alves G, Tysnes OB, Haugarvoll K, et al. Neuronal complex I deficiency occurs throughout the Parkinson’s disease brain, but is not associated with neurodegeneration or mitochondrial DNA damage. Acta Neuropathol. 2018;135:409–25. 29270838 10.1007/s00401-017-1794-7

[15] Mann VM, Cooper JM, Krige D, Daniel SE, Schapira AH, Marsden CD. Brain, skeletal muscle and platelet homogenate mitochondrial function in Parkinson’s disease. Brain. 1992;115(Pt 2):333–42. 1606472 10.1093/brain/115.2.333

[16] Gonzalez-Rodriguez P, Zampese E, Stout KA, Guzman JN, Ilijic E, Yang B, Tkatch T, Stavarache MA, Wokosin DL, Gao L, et al. Disruption of mitochondrial complex I induces progressive parkinsonism. Nature. 2021;599:650–6. 34732887 10.1038/s41586-021-04059-0 PMC9189968

[17] Tanner CM, Kamel F, Ross GW, Hoppin JA, Goldman SM, Korell M, Marras C, Bhudhikanok GS, Kasten M, Chade AR, et al. Rotenone, paraquat, and Parkinson’s disease. Environ Health Perspect. 2011;119:866–72. 21269927 10.1289/ehp.1002839 PMC3114824

[18] Samaranch L, Lorenzo-Betancor O, Arbelo JM, Ferrer I, Lorenzo E, Irigoyen J, Pastor MA, Marrero C, Isla C, Herrera-Henriquez J, Pastor P. PINK1-linked parkinsonism is associated with Lewy body pathology. Brain. 2010;133:1128–42. 20356854 10.1093/brain/awq051

[19] Pramstaller PP, Schlossmacher MG, Jacques TS, Scaravilli F, Eskelson C, Pepivani I, Hedrich K, Adel S, Gonzales-McNeal M, Hilker R, et al. Lewy body Parkinson’s disease in a large pedigree with 77 parkin mutation carriers. Ann Neurol. 2005;58:411–22. 16130111 10.1002/ana.20587

[20] Di Maio R, Barrett PJ, Hoffman EK, Barrett CW, Zharikov A, Borah A, Hu X, McCoy J, Chu CT, Burton EA, et al. Alpha-synuclein binds to TOM20 and inhibits mitochondrial protein import in Parkinson’s disease. Sci Transl Med. 2016;8:342ra378. 10.1126/scitranslmed.aaf3634 PMC5016095 27280685

[21] Paillusson S, Gomez-Suaga P, Stoica R, Little D, Gissen P, Devine MJ, Noble W, Hanger DP. Miller CCJ: alpha-synuclein binds to the ER-mitochondria tethering protein VAPB to disrupt ca(2+) homeostasis and mitochondrial ATP production. Acta Neuropathol. 2017;134:129–49. 28337542 10.1007/s00401-017-1704-z PMC5486644

[22] Devi L, Raghavendran V, Prabhu BM, Avadhani NG, Anandatheerthavarada HK. Mitochondrial import and accumulation of alpha-synuclein impair complex I in human dopaminergic neuronal cultures and Parkinson disease brain. J Biol Chem. 2008;283:9089–100. 18245082 10.1074/jbc.M710012200 PMC2431021

[23] Luth ES, Stavrovskaya IG, Bartels T, Kristal BS, Selkoe DJ. Soluble, prefibrillar alpha-synuclein oligomers promote complex I-dependent, Ca2+-induced mitochondrial dysfunction. J Biol Chem. 2014;289:21490–507. 24942732 10.1074/jbc.M113.545749 PMC4118111

[24] Martinez JH, Fuentes F, Vanasco V, Alvarez S, Alaimo A, Cassina A, Coluccio Leskow F, Velazquez F. Alpha-synuclein mitochondrial interaction leads to irreversible translocation and complex I impairment. Arch Biochem Biophys. 2018;651:1–12. 29702063 10.1016/j.abb.2018.04.018

[25] Ludtmann MHR, Angelova PR, Horrocks MH, Choi ML, Rodrigues M, Baev AY, Berezhnov AV, Yao Z, Little D, Banushi B, et al. Alpha-synuclein oligomers interact with ATP synthase and open the permeability transition pore in Parkinson’s disease. Nat Commun. 2018;9:2293. 29895861 10.1038/s41467-018-04422-2 PMC5997668

[26] Choi ML, Chappard A, Singh BP, Maclachlan C, Rodrigues M, Fedotova EI, Berezhnov AV, De S, Peddie CJ, Athauda D, et al. Pathological structural conversion of alpha-synuclein at the mitochondria induces neuronal toxicity. Nat Neurosci. 2022;25:1134–48. 36042314 10.1038/s41593-022-01140-3 PMC9448679

[27] Mahul-Mellier AL, Burtscher J, Maharjan N, Weerens L, Croisier M, Kuttler F, Leleu M, Knott GW, Lashuel HA. The process of Lewy body formation, rather than simply alpha-synuclein fibrillization, is one of the major drivers of neurodegeneration. Proc Natl Acad Sci U S A. 2020;117:4971–82. 32075919 10.1073/pnas.1913904117 PMC7060668

[28] Zhang S, Zhu R, Pan B, Xu H, Olufemi MF, Gathagan RJ, Li Y, Zhang L, Zhang J, Xiang W, et al. Post-translational modifications of soluble alpha-synuclein regulate the amplification of pathological alpha-synuclein. Nat Neurosci. 2023;26:213–25. 36690898 10.1038/s41593-022-01239-7 PMC10103650

[29] Backman CM, Malik N, Zhang Y, Shan L, Grinberg A, Hoffer BJ, Westphal H, Tomac AC. Characterization of a mouse strain expressing cre recombinase from the 3’ untranslated region of the dopamine transporter locus. Genesis. 2006;44:383–90. 16865686 10.1002/dvg.20228

[30] O’Neill B, Patel JC, Rice ME. Characterization of optically and electrically evoked dopamine release in Striatal Slices from Digenic knock-in mice with DAT-Driven expression of Channelrhodopsin. ACS Chem Neurosci. 2017;8:310–9. 28177213 10.1021/acschemneuro.6b00300 PMC5314427

[31] Costa KM, Schenkel D, Roeper J. Sex-dependent alterations in behavior, drug responses and dopamine transporter expression in heterozygous DAT-Cre mice. Sci Rep. 2021;11:3334. 33558587 10.1038/s41598-021-82600-x PMC7870653

[32] Chohan MO, Esses S, Haft J, Ahmari SE, Veenstra-VanderWeele J. Altered baseline and amphetamine-mediated behavioral profiles in dopamine transporter cre (DAT-Ires-Cre) mice compared to tyrosine hydroxylase cre (TH-Cre) mice. Psychopharmacology. 2020;237:3553–68. 32778904 10.1007/s00213-020-05635-4 PMC10120402

[33] Chen E, Lallai V, Sherafat Y, Grimes NP, Pushkin AN, Fowler JP, Fowler CD. Altered baseline and nicotine-mediated behavioral and cholinergic profiles in ChAT-Cre mouse lines. J Neurosci. 2018;38:2177–88. 29371319 10.1523/JNEUROSCI.1433-17.2018 PMC5830509

[34] Mao X, Ou MT, Karuppagounder SS, Kam TI, Yin X, Xiong Y, Ge P, Umanah GE, Brahmachari S, Shin JH et al. Pathological alpha-synuclein transmission initiated by binding lymphocyte-activation gene 3. Science 2016, 353. 10.1126/science.aah3374 PMC5510615 27708076

[35] Kam TI, Mao X, Park H, Chou SC, Karuppagounder SS, Umanah GE, Yun SP, Brahmachari S, Panicker N, Chen R et al. Poly(ADP-ribose) drives pathologic alpha-synuclein neurodegeneration in Parkinson’s disease. Science 2018, 362. 10.1126/science.aat8407 PMC6431793 30385548

[36] An JH, Su Y, Radman T, Bikson M. Effects of glucose and glutamine concentration in the formulation of the artificial cerebrospinal fluid (ACSF). Brain Res. 2008;1218:77–86. 18533132 10.1016/j.brainres.2008.04.007 PMC2547083

[37] Zampese E, Wokosin DL, Gonzalez-Rodriguez P, Guzman JN, Tkatch T, Kondapalli J, Surmeier WC, D’Alessandro KB, De Stefani D, Rizzuto R, et al. Ca(2+) channels couple spiking to mitochondrial metabolism in substantia nigra dopaminergic neurons. Sci Adv. 2022;8:eabp8701. 36179023 10.1126/sciadv.abp8701 PMC9524841

[38] Tantama M, Martinez-Francois JR, Mongeon R, Yellen G. Imaging energy status in live cells with a fluorescent biosensor of the intracellular ATP-to-ADP ratio. Nat Commun. 2013;4:2550. 24096541 10.1038/ncomms3550 PMC3852917

[39] Graves SM, Xie Z, Stout KA, Zampese E, Burbulla LF, Shih JC, Kondapalli J, Patriarchi T, Tian L, Brichta L, et al. Dopamine metabolism by a monoamine oxidase mitochondrial shuttle activates the electron transport chain. Nat Neurosci. 2020;23:15–20. 31844313 10.1038/s41593-019-0556-3 PMC7257994

[40] Guzman JN, Sanchez-Padilla J, Wokosin D, Kondapalli J, Ilijic E, Schumacker PT, Surmeier DJ. Oxidant stress evoked by pacemaking in dopaminergic neurons is attenuated by DJ-1. Nature. 2010;468:696–700. 21068725 10.1038/nature09536 PMC4465557

[41] Graves SM, Schwarzschild SE, Tai RA, Chen Y, Surmeier DJ. Mitochondrial oxidant stress mediates methamphetamine neurotoxicity in substantia nigra dopaminergic neurons. Neurobiol Dis. 2021;156:105409. 34082123 10.1016/j.nbd.2021.105409 PMC8686177

[42] Dobin A, Davis CA, Schlesinger F, Drenkow J, Zaleski C, Jha S, Batut P, Chaisson M, Gingeras TR. STAR: ultrafast universal RNA-seq aligner. Bioinformatics. 2013;29:15–21. 23104886 10.1093/bioinformatics/bts635 PMC3530905

[43] Anders S, Pyl PT, Huber W. HTSeq–a Python framework to work with high-throughput sequencing data. Bioinformatics. 2015;31:166–9. 25260700 10.1093/bioinformatics/btu638 PMC4287950

[44] Love MI, Huber W, Anders S. Moderated estimation of Fold change and dispersion for RNA-seq data with DESeq2. Genome Biol. 2014;15:550. 25516281 10.1186/s13059-014-0550-8 PMC4302049

[45] Subramanian A, Tamayo P, Mootha VK, Mukherjee S, Ebert BL, Gillette MA, Paulovich A, Pomeroy SL, Golub TR, Lander ES, Mesirov JP. Gene set enrichment analysis: a knowledge-based approach for interpreting genome-wide expression profiles. Proc Natl Acad Sci U S A. 2005;102:15545–50. 16199517 10.1073/pnas.0506580102 PMC1239896

[46] Henrich MT, Geibl FF, Lee B, Chiu WH, Koprich JB, Brotchie JM, Timmermann L, Decher N, Matschke LA, Oertel WH. A53T-alpha-synuclein overexpression in murine locus coeruleus induces Parkinson’s disease-like pathology in neurons and glia. Acta Neuropathol Commun. 2018;6:39. 29747690 10.1186/s40478-018-0541-1 PMC5946574

[47] Pancani T, Day M, Tkatch T, Wokosin DL, Gonzalez-Rodriguez P, Kondapalli J, Xie Z, Chen Y, Beaumont V, Surmeier DJ. Cholinergic deficits selectively boost cortical intratelencephalic control of striatum in male Huntington’s disease model mice. Nat Commun. 2023;14:1398. 36914640 10.1038/s41467-023-36556-3 PMC10011605

[48] Fujiwara H, Hasegawa M, Dohmae N, Kawashima A, Masliah E, Goldberg MS, Shen J, Takio K, Iwatsubo T. Alpha-synuclein is phosphorylated in synucleinopathy lesions. Nat Cell Biol. 2002;4:160–4. 11813001 10.1038/ncb748

[49] Ghanem SS, Majbour NK, Vaikath NN, Ardah MT, Erskine D, Jensen NM, Fayyad M, Sudhakaran IP, Vasili E, Melachroinou K, et al. Alpha-synuclein phosphorylation at serine 129 occurs after initial protein deposition and inhibits seeded fibril formation and toxicity. Proc Natl Acad Sci U S A. 2022;119:e2109617119. 35353605 10.1073/pnas.2109617119 PMC9169642

[50] Neumann M, Muller V, Kretzschmar HA, Haass C, Kahle PJ. Regional distribution of proteinase K-resistant alpha-synuclein correlates with Lewy body disease stage. J Neuropathol Exp Neurol. 2004;63:1225–35. 15624759 10.1093/jnen/63.12.1225

[51] Kuusisto E, Parkkinen L, Alafuzoff I. Morphogenesis of Lewy bodies: dissimilar incorporation of alpha-synuclein, ubiquitin, and p62. J Neuropathol Exp Neurol. 2003;62:1241–53. 14692700 10.1093/jnen/62.12.1241

[52] Duan W, Zhang YP, Hou Z, Huang C, Zhu H, Zhang CQ, Yin Q. Novel insights into NeuN: from neuronal marker to Splicing Regulator. Mol Neurobiol. 2016;53:1637–47. 25680637 10.1007/s12035-015-9122-5

[53] Sanz E, Yang L, Su T, Morris DR, McKnight GS, Amieux PS. Cell-type-specific isolation of ribosome-associated mRNA from complex tissues. Proc Natl Acad Sci U S A. 2009;106:13939–44. 19666516 10.1073/pnas.0907143106 PMC2728999

[54] Dryanovski DI, Guzman JN, Xie Z, Galteri DJ, Volpicelli-Daley LA, Lee VM, Miller RJ, Schumacker PT, Surmeier DJ. Calcium entry and alpha-synuclein inclusions elevate dendritic mitochondrial oxidant stress in dopaminergic neurons. J Neurosci. 2013;33:10154–64. 23761910 10.1523/JNEUROSCI.5311-12.2013 PMC3682382

[55] Fiorese CJ, Schulz AM, Lin YF, Rosin N, Pellegrino MW, Haynes CM. The transcription factor ATF5 mediates a mammalian mitochondrial UPR. Curr Biol. 2016;26:2037–43. 27426517 10.1016/j.cub.2016.06.002 PMC4980197

[56] Suzuki G, Imura S, Hosokawa M, Katsumata R, Nonaka T, Hisanaga SI, Saeki Y, Hasegawa M. Alpha-synuclein strains that cause distinct pathologies differentially inhibit proteasome. Elife 2020, 9. 10.7554/eLife.56825 PMC7406352 32697196

[57] Thibaudeau TA, Anderson RT, Smith DM. A common mechanism of proteasome impairment by neurodegenerative disease-associated oligomers. Nat Commun. 2018;9:1097. 29545515 10.1038/s41467-018-03509-0 PMC5854577

[58] Mosharov EV, Larsen KE, Kanter E, Phillips KA, Wilson K, Schmitz Y, Krantz DE, Kobayashi K, Edwards RH, Sulzer D. Interplay between cytosolic dopamine, calcium, and alpha-synuclein causes selective death of substantia nigra neurons. Neuron. 2009;62:218–29. 19409267 10.1016/j.neuron.2009.01.033 PMC2677560

[59] Pienaar IS, Elson JL, Racca C, Nelson G, Turnbull DM, Morris CM. Mitochondrial abnormality associates with type-specific neuronal loss and cell morphology changes in the pedunculopontine nucleus in Parkinson disease. Am J Pathol. 2013;183:1826–40. 24099985 10.1016/j.ajpath.2013.09.002 PMC4188170

[60] Yoon YS, You JS, Kim TK, Ahn WJ, Kim MJ, Son KH, Ricarte D, Ortiz D, Lee SJ, Lee HJ. Senescence and impaired DNA damage responses in alpha-synucleinopathy models. Exp Mol Med. 2022;54:115–28. 35136202 10.1038/s12276-022-00727-x PMC8894476

[61] Rodriguez L, Marano MM, Tandon A. Import and Export of misfolded alpha-synuclein. Front Neurosci. 2018;12:344. 29875627 10.3389/fnins.2018.00344 PMC5974333

[62] Ueda J, Uemura N, Ishimoto T, Taguchi T, Sawamura M, Nakanishi E, Ikuno M, Matsuzawa S, Yamakado H, Takahashi R. Ca(2+) -Calmodulin-calcineurin signaling modulates alpha-synuclein transmission. Mov Disord. 2023;38:1056–67. 37066491 10.1002/mds.29401

[63] Wu Q, Shaikh MA, Meymand ES, Zhang B, Luk KC, Trojanowski JQ, Lee VM. Neuronal activity modulates alpha-synuclein aggregation and spreading in organotypic brain slice cultures and in vivo. Acta Neuropathol. 2020;140:831–49. 33021680 10.1007/s00401-020-02227-6 PMC8030660

[64] Liu D, Li W, Ma C, Zheng W, Yao Y, Tso CF, Zhong P, Chen X, Song JH, Choi W, et al. A common hub for sleep and motor control in the substantia nigra. Science. 2020;367:440–5. 31974254 10.1126/science.aaz0956

[65] Yanovsky Y, Velte S, Misgeld U. Ca2 + release-dependent hyperpolarizations modulate the firing pattern of juvenile GABA neurons in mouse substantia Nigra pars reticulata in vitro. J Physiol. 2006;577:879–90. 17053035 10.1113/jphysiol.2006.117622 PMC1890382

[66] Guzman JN, Ilijic E, Yang B, Sanchez-Padilla J, Wokosin D, Galtieri D, Kondapalli J, Schumacker PT, Surmeier DJ. Systemic isradipine treatment diminishes calcium-dependent mitochondrial oxidant stress. J Clin Invest. 2018;128:2266–80. 29708514 10.1172/JCI95898 PMC5983329

[67] Poulin JF, Gaertner Z, Moreno-Ramos OA, Awatramani R. Classification of midbrain dopamine neurons using single-cell gene expression profiling approaches. Trends Neurosci. 2020;43:155–69. 32101709 10.1016/j.tins.2020.01.004 PMC7285906

[68] Kim S, Kwon SH, Kam TI, Panicker N, Karuppagounder SS, Lee S, Lee JH, Kim WR, Kook M, Foss CA, et al. Transneuronal Propagation of pathologic alpha-synuclein from the gut to the Brain models Parkinson’s Disease. Neuron. 2019;103:627–e641627. 31255487 10.1016/j.neuron.2019.05.035 PMC6706297

[69] Shahmoradian SH, Lewis AJ, Genoud C, Hench J, Moors TE, Navarro PP, Castano-Diez D, Schweighauser G, Graff-Meyer A, Goldie KN, et al. Lewy pathology in Parkinson’s disease consists of crowded organelles and lipid membranes. Nat Neurosci. 2019;22:1099–109. 31235907 10.1038/s41593-019-0423-2

[70] Harms AS, Ferreira SA, Romero-Ramos M. Periphery and brain, innate and adaptive immunity in Parkinson’s disease. Acta Neuropathol. 2021;141:527–45. 33555429 10.1007/s00401-021-02268-5 PMC7952334

[71] Hill E, Gowers R, Richardson MJE, Wall MJ. alpha-Synuclein Aggregates Increase the Conductance of Substantia Nigra Dopamine Neurons, an Effect Partly Reversed by the KATP Channel Inhibitor Glibenclamide. *eNeuro* 2021, 8. 10.1523/ENEURO.0330-20.2020 PMC7810260 33229413

[72] Liu GY, Sabatini DM. mTOR at the nexus of nutrition, growth, ageing and disease. Nat Rev Mol Cell Biol. 2020;21:183–203. 31937935 10.1038/s41580-019-0199-y PMC7102936

[73] Khan MR, Yin X, Kang SU, Mitra J, Wang H, Ryu T, Brahmachari S, Karuppagounder SS, Kimura Y, Jhaldiyal A, et al. Enhanced mTORC1 signaling and protein synthesis in pathologic alpha-synuclein cellular and animal models of Parkinson’s disease. Sci Transl Med. 2023;15:eadd0499. 38019930 10.1126/scitranslmed.add0499

[74] Johnson SC, Yanos ME, Kayser EB, Quintana A, Sangesland M, Castanza A, Uhde L, Hui J, Wall VZ, Gagnidze A, et al. mTOR inhibition alleviates mitochondrial disease in a mouse model of Leigh syndrome. Science. 2013;342:1524–8. 24231806 10.1126/science.1244360 PMC4055856

[75] Lei S, Zavala-Flores L, Garcia-Garcia A, Nandakumar R, Huang Y, Madayiputhiya N, Stanton RC, Dodds ED, Powers R, Franco R. Alterations in energy/redox metabolism induced by mitochondrial and environmental toxins: a specific role for glucose-6-phosphate-dehydrogenase and the pentose phosphate pathway in paraquat toxicity. ACS Chem Biol. 2014;9:2032–48. 24937102 10.1021/cb400894a PMC4168797

[76] Gilmozzi V, Gentile G, Castelo Rueda MP, Hicks AA, Pramstaller PP, Zanon A, Levesque M, Pichler I. Interaction of alpha-synuclein with lipids: mitochondrial cardiolipin as a critical player in the pathogenesis of Parkinson’s Disease. Front Neurosci. 2020;14:578993. 33122994 10.3389/fnins.2020.578993 PMC7573567

[77] Weber RA, Yen FS, Nicholson SPV, Alwaseem H, Bayraktar EC, Alam M, Timson RC, La K, Abu-Remaileh M, Molina H, Birsoy K. Maintaining Iron Homeostasis is the key role of lysosomal acidity for cell proliferation. Mol Cell. 2020;77:645–e655647. 31983508 10.1016/j.molcel.2020.01.003 PMC7176020

