# Supplementary material for: α-Synuclein pathology disrupts mitochondrial function in dopaminergic and cholinergic neurons at-risk in Parkinson’s disease
Source: Mol Neurodegener. 2024 Oct 8;19:69. doi: 10.1186/s13024-024-00756-2 (PMC11462807; doi:10.1186/s13024-024-00756-2)
Supplement: Supplementary file 1 — Supplementary Material 1 [file 13024_2024_756_MOESM1_ESM.pdf]

Additional file 1:

α-Synuclein pathology disrupts mitochondrial function in dopaminergic and cholinergic neurons at-risk in Parkinson's disease

Fanni F. Geibl[1,2,3*], Martin T. Henrich[1,2,3*], Zhong Xie[1], Enrico Zampese[1,10], Jun Ueda[1], Tatiana Tkatch[1, 10], David L. Wokosin[1], Elena Nasiri[2,3], Constantin A. Grotmann[2,3], Valina L. Dawson[4,5,6,7], Ted M. Dawson[4,5,6,8,10], Navdeep S. Chandel[9], Wolfgang H. Oertel[2#], D. James Surmeier[1,10#§]

Affiliations:

[1] Department of Neuroscience, Feinberg School of Medicine, Northwestern University, Chicago, IL 60611, USA.

[2] Department of Neurology, Philipps University Marburg, Marburg 35043, Germany.

[3] Department of Psychiatry and Psychotherapy, Philipps University Marburg, Marburg 35043, Germany.

[4] Neuroregeneration and Stem Cell Programs, Institute for Cell Engineering, Johns Hopkins University School of Medicine, Baltimore, MD 21205, USA.

[5] Department of Neurology, Johns Hopkins University School of Medicine, Baltimore, MD 21205, USA.

[6] Solomon H. Snyder Department of Neuroscience, Johns Hopkins University School of Medicine, Baltimore, MD 21205, USA.

[7] Department of Physiology, Johns Hopkins University School of Medicine, Baltimore, MD 21205, USA.

[8] Department of Pharmacology and Molecular Sciences, Johns Hopkins University School of Medicine, Baltimore, MD 21205, USA.

[9] Department of Medicine, Feinberg School of Medicine, Northwestern University, Chicago, IL 60611, USA

[10] Aligning Science Across Parkinson's (ASAP) Collaborative Research Network, Chevy Chase, MD 20815 US

[*]These authors contributed equally to this work.

[#]These authors contributed to this work as co-senior authors.

[§]Corresponding author: j-surmeier@northwestern.edu

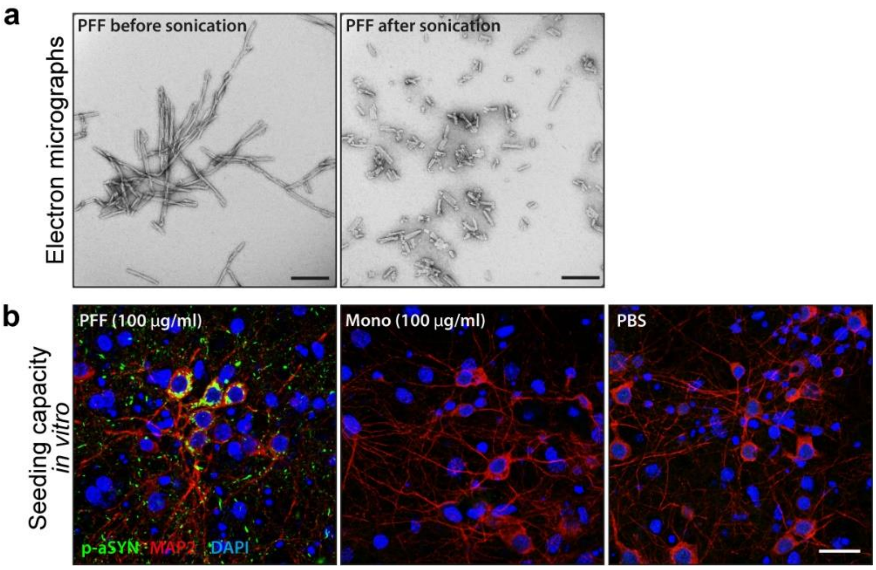

Suppl. Fig. S1 | aSYN PFF quality control data. a, Electron micrographs showing structure of aSYN PFFs before and after sonication. Scale bar 200 nm. **b,** Immunofluorescence images depicting successful seeding capacity of aSYN PFFs *in vitro* in primary neuron culture, and absence of p-aSYN signal when cultures were treated with monomeric aSYN or PBS. Scale bar 50 µm.

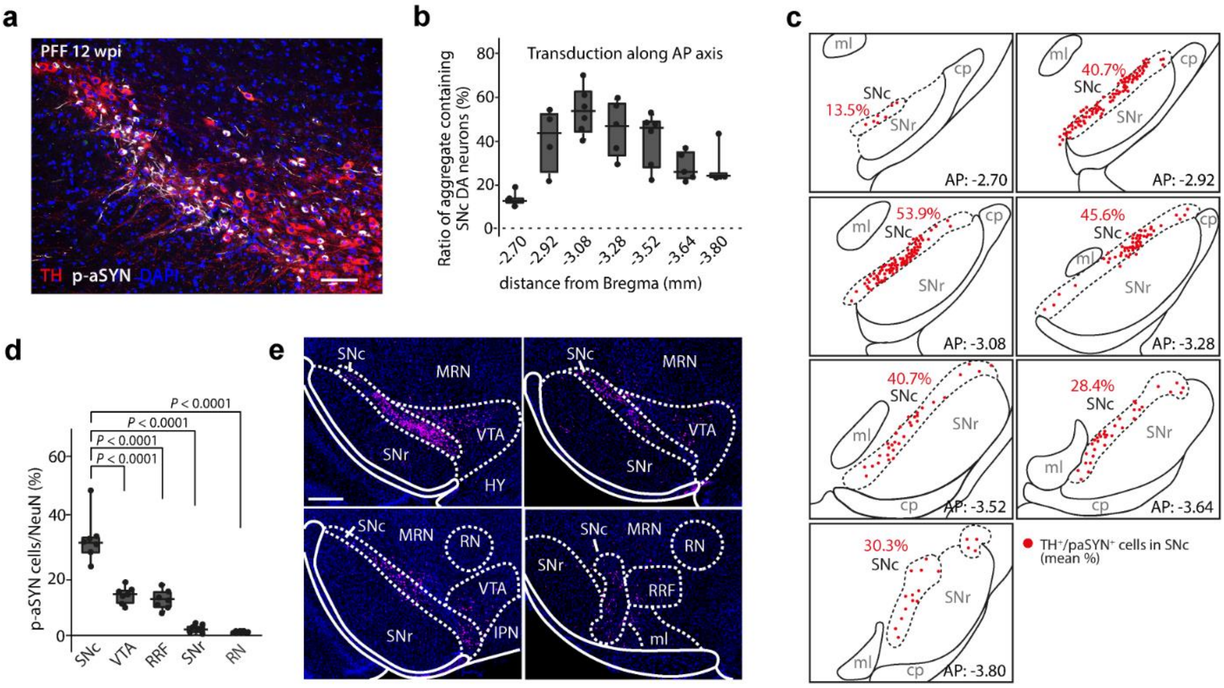

Suppl. Fig. S2 | Homogenous distribution of seeded α-synucleinopathy in DA SNc neurons of DAT-Cre-WT mice. a, TH$^+$ SNc neurons exhibiting p-aSYN pathology 12 weeks after initial seeding. Scale bar 100 µm. **b,** Box plots showing distribution of TH$^+$ SNc neurons harboring p-aSYN aggregates over the rostro-caudal extent of the SNc (box plots represent median and interquartile range, whiskers min/max value; Bregma -2.70 (*N* = 7, *n* = 3), Bregma -2,92 (*N* = 7, *n* = 4), Bregma -3.08 (*N* = 7, *n* = 6), Bregma -3.28 (*N* = 7, *n* = 5), Bregma -3.52 (*N* = 7, *n* = 6), Bregma -3.64 (*N* = 7, *n* = 5), Bregma -3.80 (*N* = 7, *n* = 3). **c,** Percentage of p-aSYN$^+$ TH$^+$ cells depicted for the analyzed AP-coordinates. **d,** Boxplot showing percentage of p-aSYN$^+$ neurons per all NeuN$^+$ neurons in the different brain regions located within the injection spot (box plots represent median and interquartile range, whiskers min/max value; (N = 7). **e,** Representative images of the local distribution of p-aSYN signal within the different brain regions. Scale bar 250 µm. Abbreviations: cp, cerebral peduncle; IPN, interpeduncular nucleus; ml, medial lemniscus; MRN, midbrain reticular nucleus; RN, red nucleus; RRF, retro rubral field; SNc, substantia nigra pars compacta; SNr, Substantia nigra pars reticulata; VTA, ventral tegmental area.

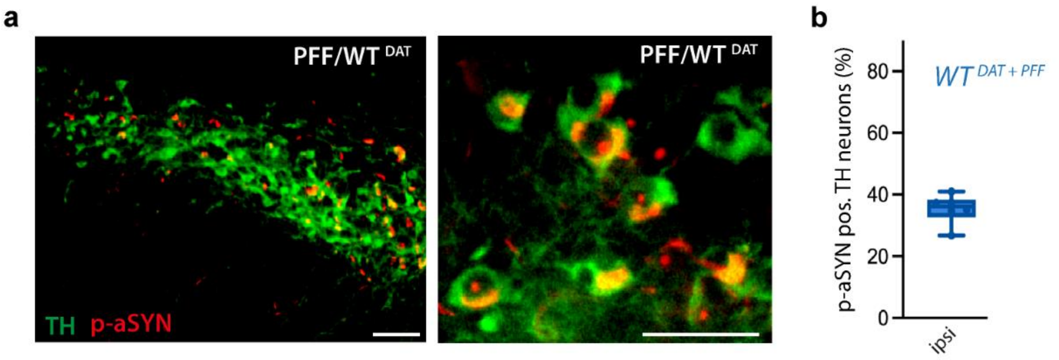

Suppl. Fig. S3 | p-aSYN pathology at 6 weeks post PFF injection. a, TH^+ DA SNc neurons exhibiting p-aSYN pathology 6 weeks after PFF injection in DAT-Cre-WT mice. Scale bar, 100 µm in overviews, 50 µm in magnified images. **b,** Transduction rate for PFF injected DAT-Cre-WT (*N* = 6; median ± min/max).

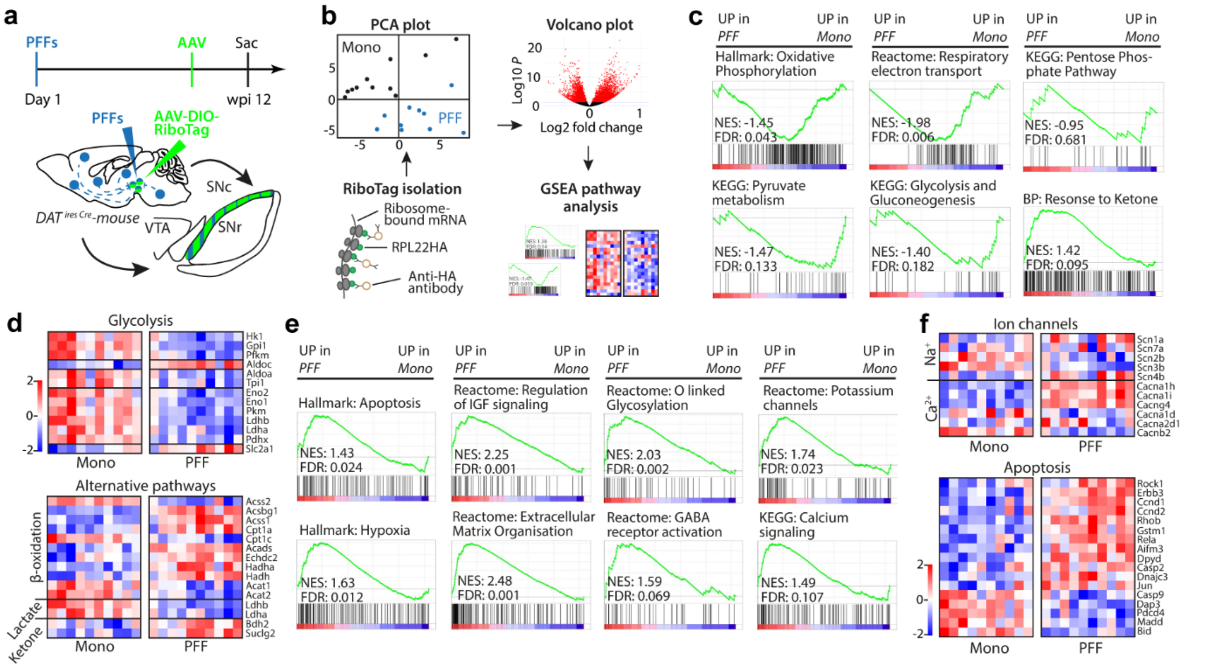

Suppl. Fig. S4 | RNAseq workflow and pathway analysis using GSEA. a, Experimental protocol. **b,** RNAseq workflow showing RiboTag isolation, quality control including PCA plot, analysis of differential gene expression, and pathway analysis using GSEA. **c,** Gene set enrichment analysis (GSEA) for metabolic related pathways in DA SNc neurons from mice injected with either aSYN PFF or monomeric aSYN (Mono (N = 10), PFF (N = 10)). **d,** Heatmaps of RNASeq analysis showing significantly down- or upregulated genes of glycolysis, β-oxidation, lactate metabolism, and ketone body consumption (Mono (N = 10), PFF (N = 10), Wald test adjusted using Benjamini-Hochberg method, $p < 0.05$). **e,** Plots depicting GSEA for highly enriched pathways in aSYN PFF injected mice (Mono (N = 10), PFF (N = 10)). **f,** Heatmaps of RNASeq analysis showing significantly down- or upregulated genes of certain ion channels and apoptosis (Mono (N = 10), PFF (N = 10), Wald test adjusted using Benjamini-Hochberg method, $p < 0.05$). Abbreviations: KEGG, Kyoto Encyclopedia of Genes and Genomes; FDR, false discovery rate. NES, normalized enrichment score; y-axis, enrichment score; x-axis, rank in ordered dataset.

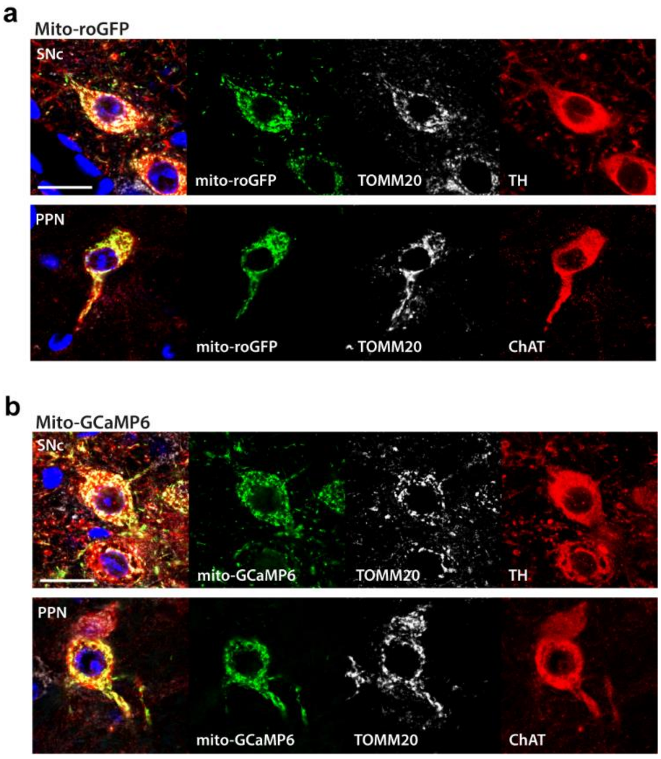

Suppl. Fig. S5 | Mitochondrial localization of mito-roGFP and mitoGCaMP6. a, Detection of mitochondrial expression of mito-roGFP, visualized by colocalization of mito-roGFP with the mitochondrial marker TOMM20 in TH+ SNc neurons and ChAT+ PPN neurons. Scale bar 25 µm. **b,** Detection of mitochondrial expression of mito-GCaMP6, visualized by colocalization of mito-GCaMP6 with the mitochondrial marker TOMM20 in TH+ SNc neurons and ChAT+ PPN neurons. Scale bar 25 µm.

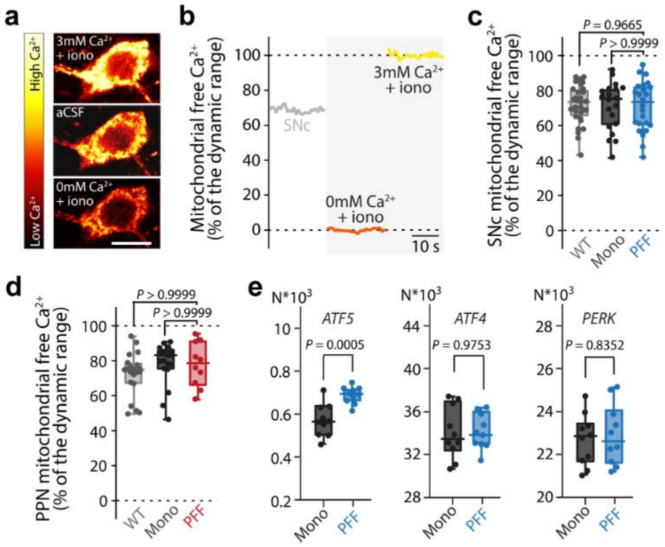

Suppl. Fig. S6 | Mitochondrial Ca^{2+}-levels and Induced Stress Response (ISR) genes. a,b, The dynamic range of the expressed mito-GCaMP6 probe was determined by the addition of the Ca^{2+}-ionophore ionomycin (iono) in aCSF with 0 mM Ca^{2+} (lowest Ca^{2+}-level) followed by the application of aCSF with 3 mM Ca^{2+} (highest Ca^{2+}-level). Scale bar 25 µm. **c,** Mitochondrial Ca^{2+}-levels in DA SNc neurons were unaffected by induced synucleinopathy (box plots represent median and interquartile range, whiskers min/max value; WT (N = 5, n = 31), Mono (N = 5, n = 20), PFF (N = 6, n = 24), One-way ANOVA test with Tukey's multiple comparisons). **d,** Mitochondrial Ca^{2+}-levels in $ChAT^{+}$ PPN neurons were also unaffected by induced synucleinopathy (box plots represent median and interquartile range, whiskers min/max value; WT (N = 5, n = 21), Mono (N = 5, n = 19), PFF (N = 5, n = 10), Kruskal-Wallis test with Dunn's multiple comparisons). **e,** Box plots depicting normalized gene expression values for ISR genes (ATF5, ATF4, PERK) highlighting upregulation of transcription factor ATF5 (box plots represent median and interquartile range, whiskers min/max value; Mono (N = 10), PFF (N = 10), Wald test adjusted using Benjamini-Hochberg method, $p < 0.05$).

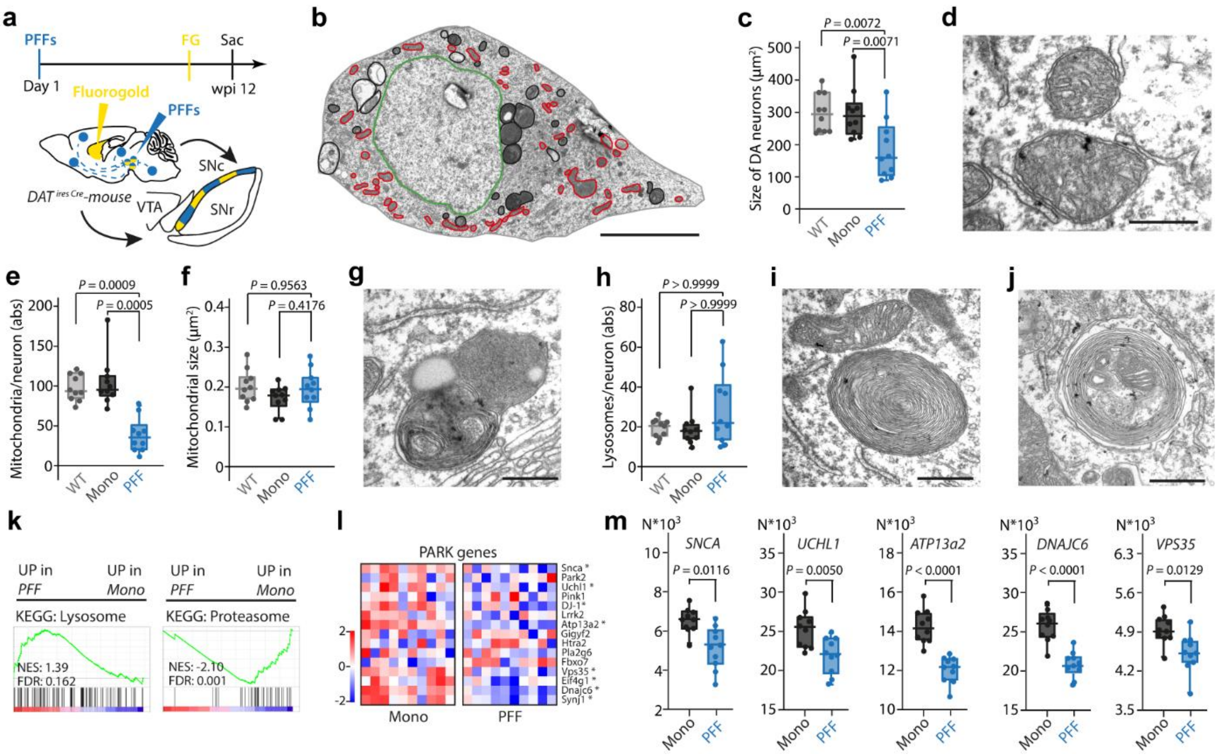

Suppl. Fig. S7 | aSYN PFF induced α-synucleinopathy causes morphological alterations of mitochondria and lysosomes. a, Experimental protocol. **b,** Transmission electron micrograph (TEM) of a SNc DA neuron from an aSYN PFF injected mouse, showing large perinuclear pathology. The nucleus is highlighted in green, mitochondria in red, and lysosomes in black, respectively. Scale bar 10 µm. **c,** Quantification of DA SNc neuronal soma size (box plots represent median and interquartile range, whiskers min/max value; WT (N = 4, n = 10), Mono (N = 4, n = 10), PFF (N = 4, n = 10), Kruskal-Wallis test with Dunn's multiple comparisons). **d,** Dysmorphic mitochondria within a DA SNc neuron from an aSYN PFF injected mouse. Scale bar 500 nm. **e,** Quantification of absolute mitochondria numbers per SNc DA neuron (box plots represent median and interquartile range, whiskers min/max value; WT (N = 4, n = 10), Mono (N = 4, n = 10), PFF (N = 4, n = 10), Kruskal-Wallis test with Dunn's multiple comparisons).**f,** Size of mitochondria per SNc DA neuron (box plots represent median and interquartile range, whiskers min/max value; WT (N = 4, n = 10), Mono (N = 4, n = 10), PFF (N = 4, n = 10), One-way ANOVA test with Tukey's multiple comparisons). **g,** TEM depicting activated lysosome within a DA SNc neuron from an aSYN PFF injected mouse. Scale bar 500 nm. **h,** Quantification of absolute lysosome numbers per DA SNc neuron (box plots represent median and interquartile range, whiskers min/max value; WT (N = 4, n = 10), Mono (N = 4, n = 10), PFF (N = 4, n = 10), Kruskal-Wallis test with Dunn's multiple comparisons). **i, j,** TEM images from SNc DA neurons from aSYN PFF injected mice depicting a lamellar body accompanied by a mitochondrion with swollen cristae structure (**i**), and a lamellar body with a trapped mitochondrion. Scale bars 500 nm. **k,** Gene set enrichment

analysis (GSEA) of DA SNc neurons from DAT-Cre mice injected with either aSYN PFF or monomeric aSYN. KEGG pathway "Lysosome" was upregulated in aSYN PFF injected mice, while KEGG pathway "Proteasome" was strongly downregulated (Mono (N = 10), PFF (N = 10). KEGG, Kyoto Encyclopedia of Genes and Genomes; FDR, false discovery rate. NES, normalized enrichment score; y-axis, enrichment score; x-axis, rank in ordered dataset. **l,** Heatmap of RNASeq analysis showing expression of PARK genes (Mono (N = 10), PFF (N = 10), Wald test adjusted using Benjamini-Hochberg method, $p < 0.05$). **m,** Box plots showing normalized gene expression values for significantly changed PARK genes (SNCA, UCHL1, ATP13a2, DNAJC6, VPS35). Note, all genes, except for SNCA, are implicated in lysosomal or proteasomal degradation of proteins (box plots represent median and interquartile range, whiskers min/max value; Mono (N = 10), PFF (N = 10), Wald test adjusted using Benjamini-Hochberg method, $p < 0.05$).

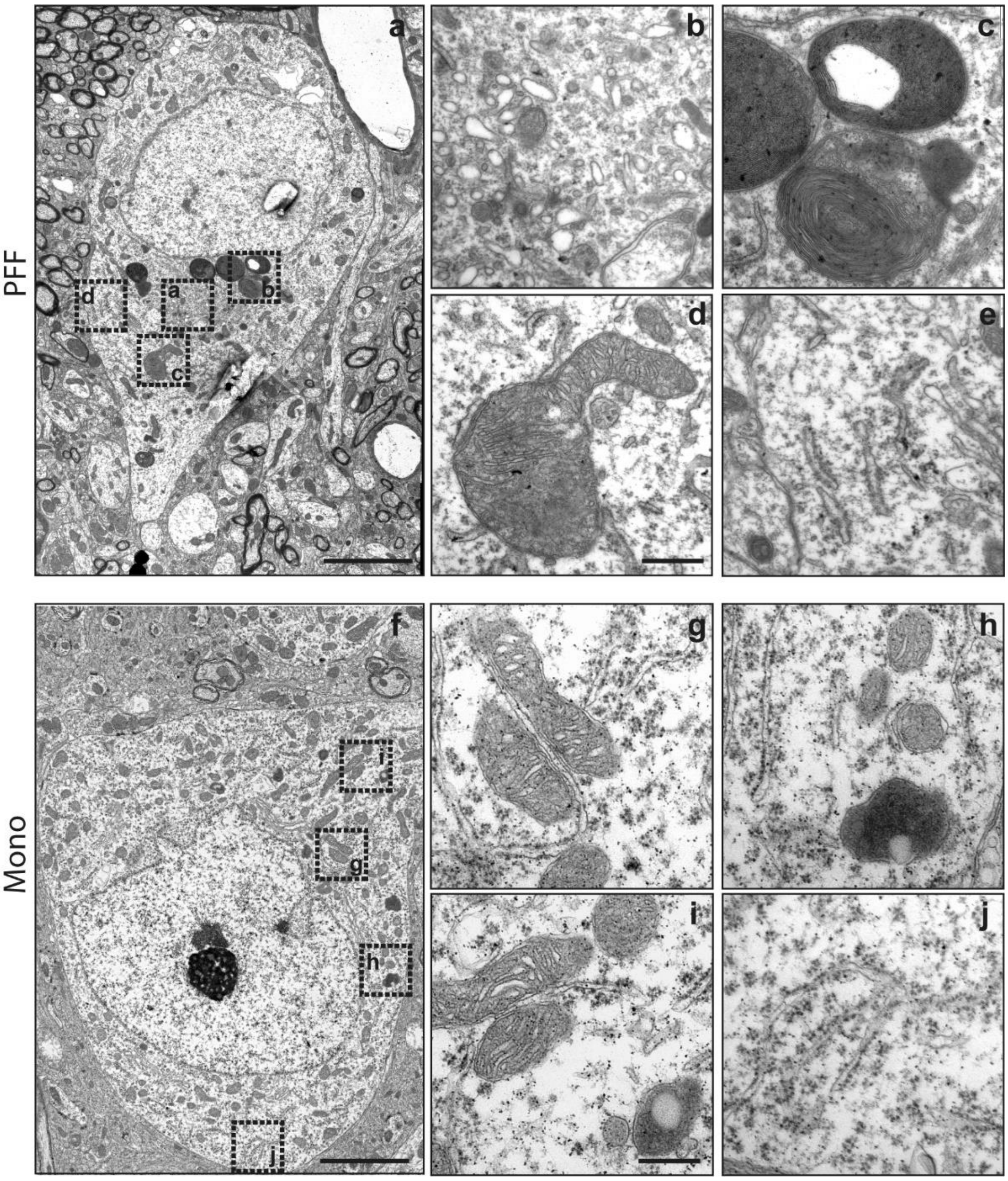

Suppl. Fig. S8 | TEM of cellular organelles in monomeric aSYN and PFF injected mice. a, Overview image showing a DA SNc neuron from an aSYN PFF injected mouse containing large perinuclear aggregate pathology. **b,** Perinuclear area composed of a crowded environment containing tubulovesicular structures, proteinaceous material, mitochondria, and small lysosomal structures. **c,** Large electron dense lysosomes exhibiting lamellar structure. **d,** Swollen mitochondrion with disrupted cristae structure. **e,** Rough endoplasmic reticulum. **f,** Overview image showing a DA SNc neuron from a monomeric aSYN injected mouse, lacking perinuclear pathology. **g,** Perinuclear area lacking crowded environment showing mitochondria. **h,** Electron dense lysosome of regular size and structure. **i,** Image depicting mitochondria with regular cristae structure. **j,** Rough endoplasmic reticulum. Scale bar in both overview images **a, f,** 10 µm. Scale bar in high magnified images 500 nm.

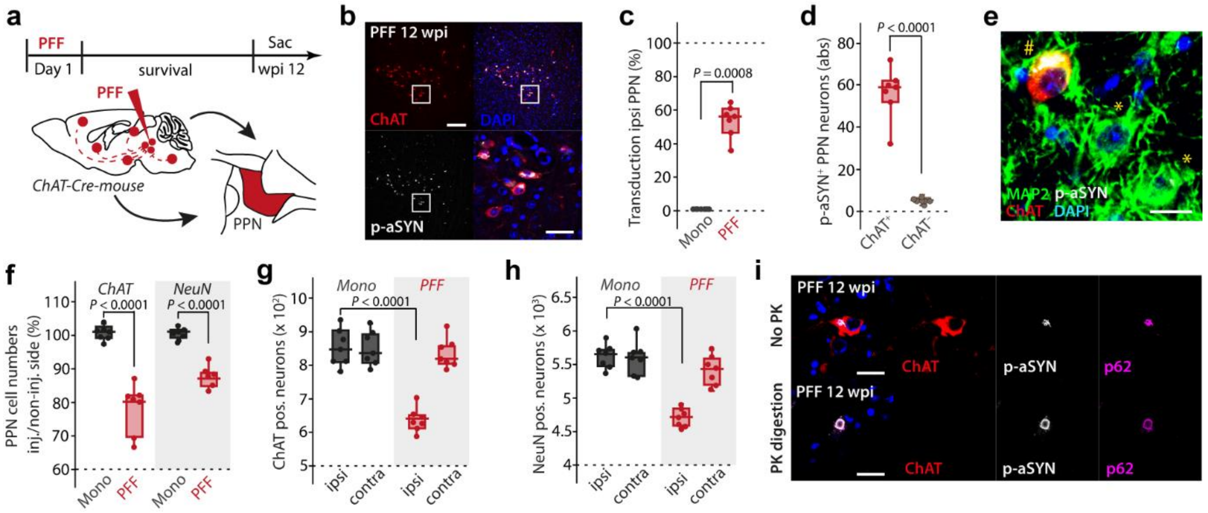

Suppl. Fig. S9 | aSYN PFF injection causes PD-like neurodegeneration of PPN cholinergic neurons. a, Experimental protocol. **b,** ChAT⁺ PPN neurons exhibiting p-aSYN pathology 12 weeks after initial seeding. Scale bar, 250 µm in overview, 50 µm in magnified image. **C,** Transduction rate indicating percentage of p-aSYN⁺ ChAT⁺ PPN neurons on the ipsilateral side of PFF injection (box plots represent median and interquartile range, whiskers min/max value; Mono (N = 7), PFF (N = 7), Mann-Whitney-U test). **d,** Box plots showing the distribution of p-aSYN⁺ cholinergic (ChAT⁺) and non-cholinergic (ChAT⁻) PPN neurons. Box plots show absolute numbers of p-aSYN⁺ neurons (box plots represent median and interquartile range, whiskers min/max value; Mono (N = 7), PFF (N = 7), Mann-Whitney-U test). **E,** p-aSYN⁺ inclusion in a MAP2⁺ ChAT⁺ PPN neuron (#) and absence of pathology in MAP2⁺ but ChAT⁻ PPN neurons (*). Scale bar 25 µm. **f,** Quantification of ChAT⁺ and NeuN⁺ PPN cells expressed as percentage of the non-injected side (box plots represent median and interquartile range, whiskers min/max value; Mono (N = 7), PFF (N = 7), Unpaired t-test). **g,** Box plots showing number of ChAT⁺ neurons in PPN (box plots represent median and interquartile range, whiskers min/max value; Mono (N = 7), PFF (N = 7), One-way ANOVA test with Dunnett's multiple comparisons). **h,** Box plots showing number of NeuN⁺ neurons in PPN (box plots represent median and interquartile range, whiskers min/max value; Mono (N = 7), PFF (N = 7), One-way ANOVA test with Dunnett's multiple comparisons). **i,** p-aSYN⁺ aggregates in ChAT⁺ PPN neurons were p62⁺ and resistant to digestion with Proteinase K (PK). Scale bar, 25 µm.

	Host	Cat. No.	Manufacturer	Dilution
**Primary antibodies**				
Tyrosine Hydroxylase	Rabbit	AB152	Merck Millipore	1:1000
Tyrosine Hydroxylase	Sheep	AB1542	Merck Millipore	1:1000
Choline Acetyltransferase (ChAT)	Goat	AB144P	Merck Millipore	1:100
Neuron N (NeuN)	Mouse	MAB377	Merck Millipore	1:1000
Alpha-synuclein (p-S129)	Rabbit	ab51253	Abcam	1:2000
SQSTM1/p62	Mouse	ab56416	Abcam	1:2000
MAP2	Chicken	ab5392	Abcam	1:2000
Green fluorescent protein (GFP)	Chicken	AB16901	Merck Millipore	1:1000
**Secondary antibodies**				
Anti-goat AlexaFluor488	Donkey	A-11055	Invitrogen	1:1000
Anti-chicken AlexaFluor488	Donkey	703-545-155	Jackson ImmunoResearch	1:1000
Anti-chicken Cy3	Donkey	703-165-155	Jackson ImmunoResearch	1:1000
Anti-goat Cy3	Donkey	705-165-147	Jackson ImmunoResearch	1:1000
Anti-mouse AlexaFluor488	Donkey	A-2102	Invitrogen	1:1000
Biotinylated anti-rabbit	Donkey	711-065-152	Jackson ImmunoResearch	1:1000
Biotinylated anti-mouse	Donkey	715-065-151	Jackson ImmunoResearch	1:1000
Streptavidin AlexaFluor647	Donkey	016-600-084	Jackson ImmunoResearch	1:1000

Supplementary Table 1 Primary and secondary antibodies used in the study
